# Genetics and Evolution of the *Salmonella* Galactose-Initiated Set of O Antigens

**DOI:** 10.1371/journal.pone.0069306

**Published:** 2013-07-18

**Authors:** Peter R. Reeves, Monica M. Cunneen, Bin Liu, Lei Wang

**Affiliations:** 1 School of Molecular Bioscience, University of Sydney, Sydney, Australia; 2 TEDA School of Biological Sciences and Biotechnology, Nankai University, TEDA, Tianjin, P. R. China; Institut National de la Recherche Agronomique, France

## Abstract

This paper covers eight *Salmonella* serogroups, that are defined by O antigens with related structures and gene clusters. They include the serovars that are now most frequently isolated. Serogroups A, B1, B2, C2-C3, D1, D2, D3 and E have O antigens that are distinguished by having galactose as first sugar, and not *N*-acetyl glucosamine or *N-*acetyl galactosamine as in the other 38 serogroups, and indeed in most *Enterobacteriaceae*. The gene clusters for these galactose-initiated appear to have entered *S. enterica* since its divergence from *E. coli*, but sequence comparisons show that much of the diversification occurred long before this. We conclude that the gene clusters must have entered *S. enterica* in a series of parallel events. The individual gene clusters are discussed, followed by analysis of the divergence for those genes shared by two or more gene clusters, and a putative phylogenic tree for the gene clusters is presented. This set of O antigens provides a rare case where it is possible to examine in detail the relationships of a significant number of O antigens. In contrast the more common pattern of O-antigen diversity within a species is for there to be only a few cases of strains having related gene clusters, suggesting that diversity arose through gain of individual O-antigen gene clusters by lateral gene transfer, and under these circumstances the evolution of the diversity is not accessible. This paper on the galactose-initiated set of gene clusters gives new insights into the origins of O-antigen diversity generally.

## Introduction

Bacterial surface polysaccharides are among the most variable structures in Biology. They occur in a variety of capacities, including as O antigens of lipopolysaccharide (LPS), capsules, teichoic acids, and protein-associated structures. Most of the diversity within bacterial species arises from major polymorphisms in which, for example, the capsule or O antigen has multiple alternative structures. Closely related genera may share some of the structures, and for example 23 of the 46 *Salmonella* O antigens are identical or very similar to an *Escherichia coli* O antigen and these pairs of O antigens have identical or very similar gene clusters (unpublished data). These gene clusters are thought to have been in their common ancestor, but there are few or none shared between more distantly related genera, such as *Yersinia* and *Escherichia* . The multiple structures in a species are usually very diverse, with variability in the sugars present, their order, and their linkages, such that the diversity cannot be attributed to evolutionary changes within the gene clusters during species divergence. Instead, structure diversification within a species appears to involve mostly gain of new structures by lateral transfer of the appropriate gene clusters, presumably balanced over time by loss of others. Thus the diversity found in a group such as *Salmonella* is assumed to have originated in a range of species and, given the enormous diversity of the Bacteria, it is not surprising that the source strains have generally not been identified. In this paper we analyse in detail a set of 8 *Salmonella enterica* O antigens, being those of serogroups A, B1, B2, C2-C3, D1, D2, D3 and E, which are distinctive in several ways that provide major insights into the evolution of O-antigen diversity. They are referred to here as the galactose- (Gal-) initiated set of O antigens.

### Historical review of the Salmonella Serotyping Scheme

The classic work of Kauffmann and White (KW) established a typing scheme for *Salmonella* based on serology of the O and H antigens [1]. The scheme has since been expanded and refined, and is maintained by the WHO Collaborating Center for Salmonella Research. The 2007 9th edition of the scheme [2] (www.pasteur.fr/sante/clre/cadrecnr/salmoms/WKLM_2007.pdf), includes 46 O antigens and 114 H antigens in 2557 serovars, each with a unique combination of O antigen and H antigens (encoded at two loci as discussed below). *Salmonella* is divided into 2 species, *S. enterica* and *S. bongori*, and *S. enterica* is divided into six subspecies: *S. enterica* subsp. *enterica* , *S.* enterica subsp. *salamae* , *S.* enterica subsp. *arizonae* , *S. enterica* subsp. *diarizonae* , *S.* enterica subsp. *indica* , and *S. enterica* subsp. *houtenae* , or aubspecies I, II, IIIa, IIIb, IV, and VI respectively. These species and subspecies are distinguished by biochemical differences. An additional subspecies was identified by sequence variation in housekeeping genes and named subspecies 7 [3], but it is not recognized in the serotyping scheme, as it is distinguished from serovar 4 only by sequence data that is not available for most isolates. The distribution of serogroups among the species and subspecies is also given in the 2007 report on the serovars [2].

The variation in O antigens is mostly due to variation among the gene clusters, which in *S. enterica* and related species maps to a single specific locus between the *galF* and *gnd* genes. In *S. enterica* it is variation in this locus that is used to define serogroups, but there are often other genes located elsewhere on the chromosome, sometimes within prophage genomes, that can also be involved in O-antigen synthesis. These additional genes are generally not essential for O-antigen incorporation into LPS, and carry out functions such as adding a side-branch glucose residue or O-acetyl groups that may not be present on all repeat units. The additions are sometimes referred to as decorations on the O antigen.

A second antigen, the H antigen, is used in combination with the O antigen to define serovars. The epitopes of the H antigen are on flagellin, the major protein of the flagellum. *Salmonella* has two loci for flagellin, *fliC* present in essentially all strains, and *fljB* present only in some subspecies [4], where it confers an alternative second H antigen. Serotyping involves the use of all three O- and H-antigen loci, giving multiple serovars, each with a unique combination. The three loci are equally important in determining the serovar, but there is a convention of using O antigens to define “serogroups” usually simply referred to as groups, which are then “divided” into serovars based on H antigens present. Serovars commonly appear to be clones with each presumably having a single origin. Serotyping of *S. enterica* has been very useful for diagnosis and epidemiology, and for this reason there is good documentation of the distribution of serovars, and retention of a range of strains for study.

As the KW scheme was refined over time, some of the better studied of the original groups were subdivided on serological grounds, e.g. group C into C1, C2 and C3. However, Le Minor, and then Popoff, and later Grimont and Weill, all at the WHO Collaborating Centre on Salmonella in Paris, adopted the principle that a serogroup should be defined by the variation at the major O-antigen locus, with each group having a specific gene cluster at the *galF*/*gnd* locus. Variation due to genes outside of that locus is treated as variation within the group (Weill, personal communication). For example, the original group C that was divided into C1, C2 and C3 on serological grounds, is now treated as comprising two groups, C1 and C2-C3, as the C1 gene cluster is quite different from those of C2 and C3, which are identical. The difference between the old C2 and C3 groups is due to genes mapping outside of the main gene cluster in a prophage genome. This new system has generally worked well, as the gain or loss of prophages occurs very readily.

### Repeat units of the *Salmonella* Galactose-initiated set of O antigens

O antigens are repeat-unit polysaccharides and in *S. enterica* all but one (O54) are synthesized by the Wzx/Wzy pathway [5] in which the repeat unit is first synthesized on a lipid carrier, undecaprenyl pyrophosphate (Und-PP), and then polymerized to form the full O-antigen polymer. The first step in this pathway is addition of a sugar phosphate to undecaprenyl phosphate (Und-P) to give the undecaprenyl pyrophosphate-linked sugar. The first sugar is galactose for the O antigens under discussion. The first O-unit sugar is a major distinguishing feature between these groups, those with GlcNAc or GalNAc being initiated by WecA, which is also the initial transferase (IT) for many O antigens in the *Enterobacteriaceae*, including most of those in *E. coli* and several other related genera such as *Citrobacter* and *Yersinia* . WecA initiates synthesis of the enterobacterial common antigen (ECA) and O-antigen repeat unit synthesis by the transfer of GlcNAcP from UDP-GlcNAc to Und-P to give Und-PP-GlcNAc. Where GalNAc is the first sugar, WecA forms Und-PP-GlcNAc as usual, which is then converted to Und-PP-GalNAc [6]. The *wecA* gene is located within the gene cluster for ECA, which is generally present in the *Enterobacteriaceae.*

WbaP is responsible for Und-PP-Gal formation in the Gal-initiated O antigens, but the *wbaP* gene is otherwise uncommon, being reported only in the capsule gene clusters of *E. coli* K30 and *Klebsiella pneumoniae* K20 [7,8]. We looked for other homologues by using *S. enterica* WbaP in a BLASTp search against the non-redundant protein sequences database (www.blast.ncbi.nlm.gov.nih /), using the BLOSUM62 matrix, a gap creation penalty of 11, and gap extension penalty of 1, and identified homologues in *Citrobacter* *koseri* , *Pectobacterium* *atrosepticum* , *P* *. wasabiae* , and several *Erwinia* and *Pantoea* species, for which the analysis of adjacent sequence data suggest that these homologues are in polysaccharide gene clusters (data not shown)*.*

It seems clear that the ancestral situation in *Salmonella* is initiation by WecA with GlcNAc or GalNAc as first sugar, as these sugars are widespread in the *Enterobacteriaceae*, and for most species there are only sporadic occurrences of initiation by an IT other than WecA. It appears that the use of Gal as initial sugar appeared in *Salmonella* since its divergence from *Escherichia* , but the eight Gal-initiated O antigens have grown to be very important in this species. For example, they are present in about 94% of those that could be typed from the *Salmonella* isolates listed by the USA Centers for Diseases Control for 1999 to 2000 (http://www.cdc.gov/ncezid/dfwed/PDFs/SalmonellaAnnualSummaryTables2009.pdf).

**Figure 1:**
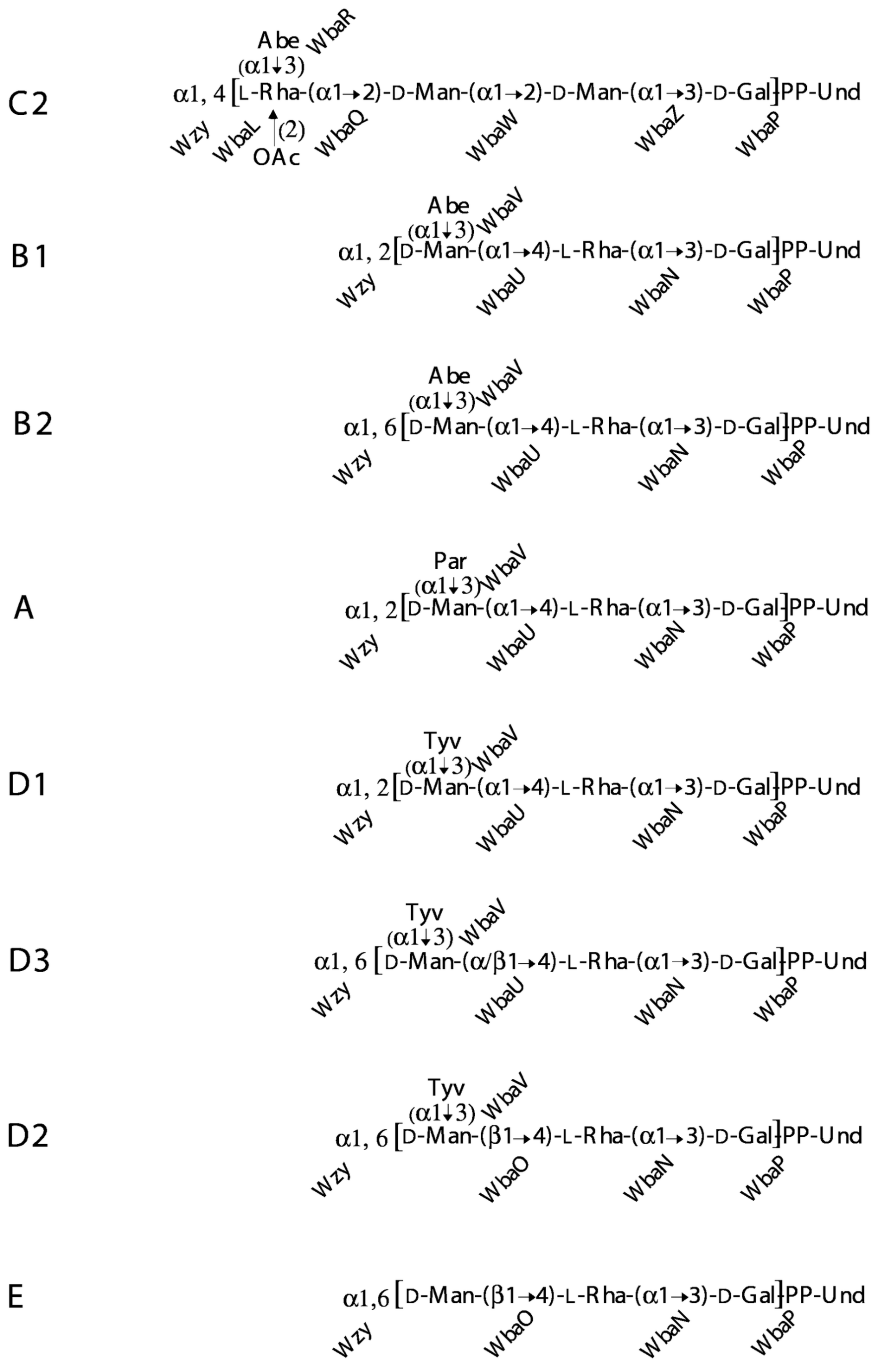
Structures of the Galactose-initiated O units of *S. enterica*. A single O unit is shown as it is built on the lipid carrier, Und-PP. The transferase responsible for the addition of each sugar of the O unit is shown. The repeat unit is given in square brackets. The Wzy polymerisation linkage is also shown to the left of the repeat unit. Sugar abbreviations: Abe, abequose; Gal, galactose; Man, mannose; Par, paratose; Rha, rhamnose; Tyv, Tyvelose.

The *S. enterica* Gal-initiated O antigens have many structural similarities in addition to having Gal as the initiating sugar (Figure 1). Although mostly very common, they are found almost exclusively in subspecies 1 and 2, with the only exception among the type strains being two C2-C3 serovars in subspecies 3b [2]. None have been found in *S. bongori*. There are now several gene-cluster sequences available for most of the eight groups, either sequenced as gene clusters or derived from sequenced genomes, and within each group these agree on gene content and gene order, with the exception of an additional *orf* in some group E strains. We have generally used one representative for each O antigen for comparisons, using duplicate sequences only as required (see Table 1 for details of sequences used in this study).

**Table 1:** *S. enterica* strains and sequences used in this study.

Group^1^,^2^	Subspecies	Serovar	Strain Name	Accession No.	Sequence positions *rmlB-wbaP* *	Source sequence description
C2^1^	I	Newport	SL254	CP001113	complement (2169633.2190020)	genome
C2	I	Hadar	RI_05P066	NZ_ABFG01000001	143474.163861	genome
C2	I	Newport	SL317	NZ_ABEW01000006	350091.370478	genome
C2	I	Kentucky	CVM29188	NZ_ABAK02000001	complement (1547806.1568193)	genome
C2	I	Kentucky	CDC 191	NZ_ABEI01000001	complement (71706.92093)	genome
C2	I	Muenchen	M67	X61917	-	*abe* to end of *wbaP*
B1^1^	I	Heidelberg	SL476	CP001120	complement (2208550.2226199)	genome
B1	I	Agona	SL483	CP001138	complement (2118797.2136446)	genome
B1	I	Typhimurium	LT2	AE006468	complement (2160931.2178580)	genome
B1	I	Paratyphi B	SPB7	CP00886	867219.884868	genome
B1	I	4{5}, ,12:i: -	CVM23701	ABAO01000005.1	complement (64717.82366)	genome
B1	I	Saintpaul	SARA23	NZ_ABAM02000001	complement (2620030.2637679)	genome
B1	I	Saintpaul	SARA29	NZ_ABAN01000008	complement (25684.43334)	genome
B1	I	Typhimurium	LT2 SL1654	X56793	-	*wcaL* to start of *gnd*
B2^1^	I	Schwarzengrund	CVM19633	CP001127	complement (2180209.2200123)	genome
B2	I	Schwarzengrund	SL480	NZ_ABEJ01000005	complement (34745.54614)	genome
A^2^	I	Paratyphi A	AKU_12601	FM200053	861093.878865	genome
A^2^	I	Paratyphi A	ATCC 9150	CP000026	860063.884690	genome
D1^1^	I	Dublin	CT_02021853	CP001144	complement (2291376.2310233	genome
D1	I	Javiana	GA_MM04042433	NZ_ABEH02000005	complement (80603.99461)	genome
D1	I	Typhi	Ty2	AE014613	867359.886216	genome
D1	I	Enteritidis	P125109	AM944172	complement (2164360.2183216)	genome
D1	I	Gallinarum	287/91	AM933173	complement (2155362.2174224)	genome
D1	I	Typhi	Ty2	M29682	-	*prt-tyv*
D1	I	Typhi	Ty2	M65054	-	*wzx-wbaV*
D3^1^	II	1,9,12,46,27:c:z39	M1168	KC688886		*galF* to *gnd*
D3	II	1,9,12,46,27:c:z39	M1168	AF017148	-	end of *wbaV* to *wzy*
D2^1^	I	Strasbourg	M388	KC688887	-	*galF* to *gnd*
D2	I	Strasbourg	M388	U04164	-	*wbaN* (partial)
D2	I	Strasbourg	M388	U04165	-	end of *wbaV* to start of *wbaO*
E1	I	Weltevreden	HI_N05-537	NZ_ABFF01000001	450487.464862	incomplete genome
E1^1^	I	Anatum	M32	KC688885		*galF* to *gnd*
E1	I	Anatum	M32	X60665(new)/ S93643 (old)	-	end *rmlD* to start of *manC*
E1	I	Anatum	M32	X60666(new)/ S93671 (old)	-	orf17.4 to start of *gnd*
E4^1^	I	Senftenberg	M227	KC688884		*galF* to *gnd*
B1^2^	I	Azteca	M1996	AY062292	-	*wbaV-wbaU* intergenic region
B2^2^	I	Schleissheim	M1412	AY062284	-	*wbaV-wbaU* intergenic region
B2^2^	I	Sloterdijk	M2105	AY062287	-	*wbaV-wbaU* intergenic region
B1^2^	I	Tejas	M2110	AY062289	-	*wbaV-wbaU* intergenic region
B1^2^	I	Tennyson	M2111	AY062290	-	*wbaV-wbaU* intergenic region
B1^2^	I	Texas	M2112	AY062291	-	*wbaV-wbaU* intergenic region
B1^2^	I	Travis	M2072	AY062288	-	*wbaV-wbaU* intergenic region
B2^2^	I	Vellore	M2120	AY062285	-	*wbaV-wbaU* intergenic region
B1^2^	II	4,12,27:i:z35	M2041	AY062286	-	*wbaV-wbaU* intergenic region
B1^2^	II	4,12:z:1,7	M2019	AY062293	-	*wbaV-wbaU* intergenic region

1 Group representatives used for sequence analysis.

2 Referred to in figures

* positions given for genome sequences only.

### Synthesis and processing of O antigens

The synthesis of O antigens, and LPS in general, has been reviewed quite often [5,9–11], and will be only briefly described here using the group B1 strain LT2 as an example. Strain LT2 has been used extensively as a model experimental organism, and as a consequence, the B1 O-antigen gene cluster was the first to be described [12] and later sequenced [13]. All of the nucleotide-sugar biosynthetic pathways are known [14–17], as are the glycosyl transferase functions [18–21], and the processing steps have also been characterised [22–26].

**Figure 2:**
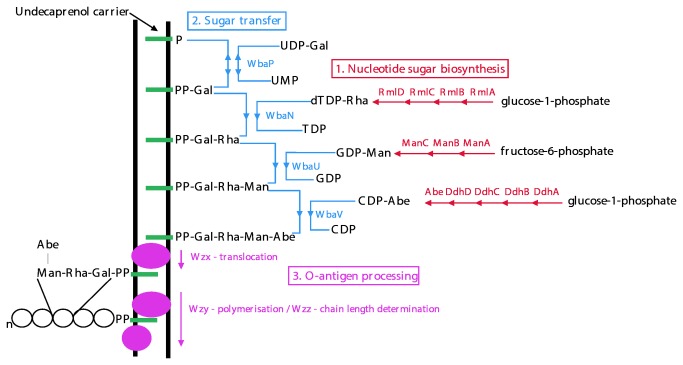
Synthesis of the *S. enterica* group B O antigen. The three stages of O-unit synthesis are shown: 1. Nucleotide-sugar biosynthesis (red), 2. O-unit assembly (blue), and 3. O-unit processing (purple). The proteins involved at each step are indicated. The final polymerised O-antigen chain is indicated by circles, where n indicates a variable number of repeat units present. Modified from [9]. Sugar abbreviations: Abe, abequose; Gal, galactose; Man, mannose; Rha, rhamnose.

**Figure 3:**
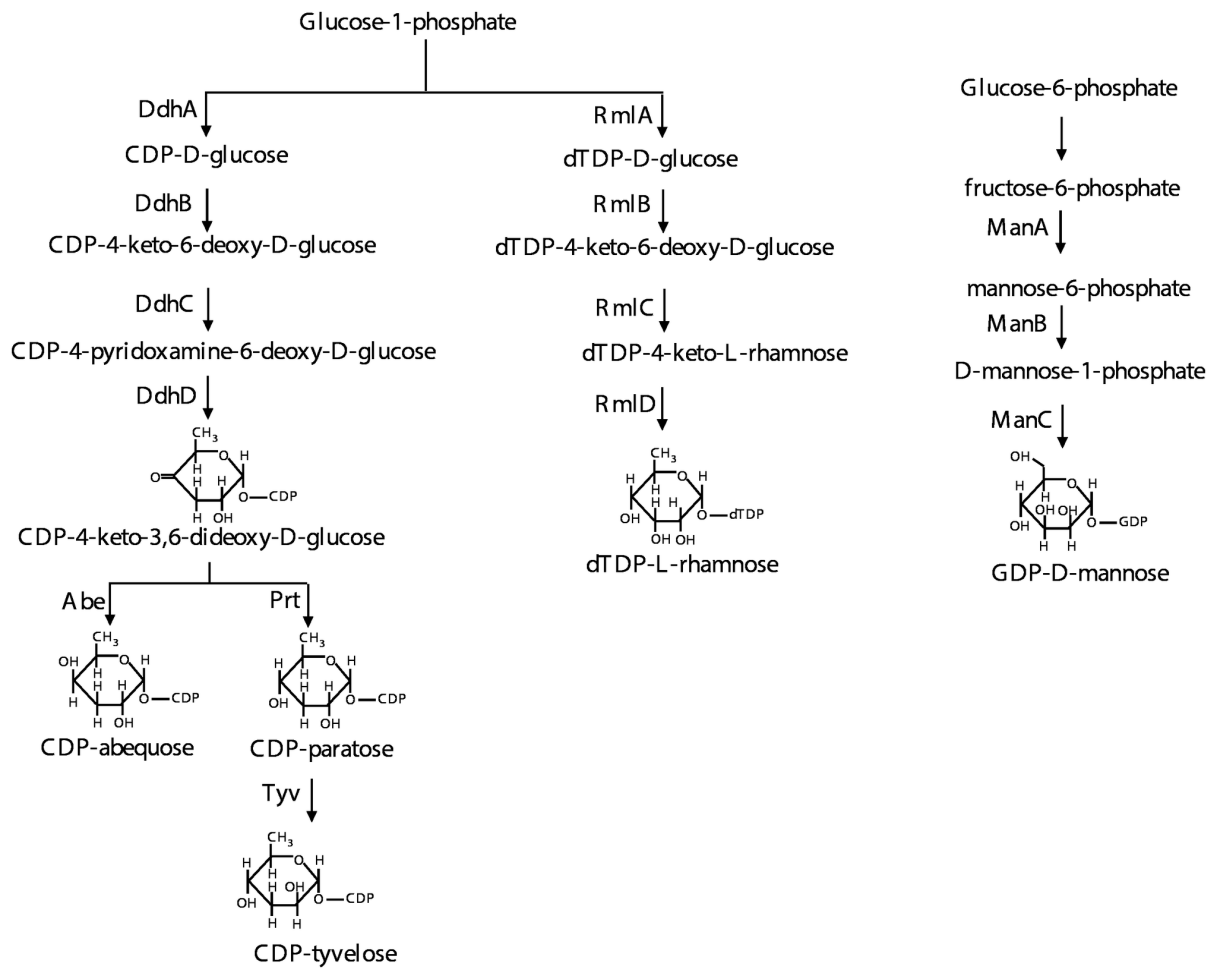
Biosynthesis of O-antigen-related NDP-linked sugars. Enzymes involved at each step are indicated. Sugar structures are shown for the end products and for one branch point intermediate.

The biosynthetic pathway for B1, summarised in Figure 2, includes genes for synthesis of the nucleotide-diphosphate (NDP) -sugar precursors: CDP-abequose, dTDP-rhamnose and GDP-mannose, but not those for UDP-galactose which are encoded elsewhere. Figure 3 gives the details of these pathways. Once Group B1 O-unit formation has been initiated on the cytoplasmic face of the cell membrane by WbaP, the rhamnose, mannose and abequose sugars are then transferred sequentially from their NDP-sugar precursors by a series of glycosyl transferases (GTs) to give the completed repeat unit (Figure 2).

The O unit is then translocated (flipped) across the inner membrane by a translocase known as Wzx. Once in the periplasm, most repeat units are polymerised by Wzy to give long-chain length O antigen, still on the Und-PP carrier lipid. O antigen is then ligated to the lipid A-core by WaaL, which is encoded in the LPS core gene cluster. The completed LPS molecule is then exported to the outer leaflet of the outer membrane. There is always a substantial proportion of LPS that is exported without O antigen, and the remainder will have O antigen with one or more repeat units. Note that Wzy determines the details of the polymerization linkage, which accounts in part for the diversity of *wzy* genes. Determination of the number of repeat units in each chain is complex and the mechanism is not understood, but Wzz, encoded just outside the major gene cluster plays a major role (reviewed in 11).

In this study we have taken the published work on the galactose-initiated O antigens and added new sequence data to give full gene-cluster sequences for each group, which provides the data for comparing the gene clusters and analysis of the evolutionary significance of the relationships observed. Details of the strains involved are given in Table 1.

## Materials and Methods

### Construction of DNase I shotgun banks, DNA sequencing and data analysis

Chromosomal DNA was prepared as previously described [27]. Primers WL_1098 (5′-ATTGGTAGCTGTAAGCCAAGGGCGGTAGCGT-3′) and WL_2211 (5′-CACTGCCATACCGACGACGCCGATCTGTTGCTTGG-3′) [28], based on sequences from the JUMPStart site and *gnd* genes, respectively, were used to amplify O-antigen gene clusters of *Salmonella* groups D2, D3, E1 and E4 using the Expand Long Template PCR system from Roche. The PCR cycle used was as follows: denaturation at 94 °C for 10 s, annealing at 60 °C for 30 s and extension at 68 °C for 15 min. The PCR products were digested with DNase I and the resulting DNA fragments were cloned into pGEM-T easy (Promega) to produce a bank using the method described previously [28]. DNA sequencing was carried out by the Tianjin Biochip Corporation, using an ABI 3730 automated DNA sequencer. Sequence data were assembled and analyzed as described previously [29]. The sequences have been deposited in GenBank with accession numbers shown in Table 1.

## Results and Discussion

### Overview of the Gene Clusters of the Gal-initiated O antigens

**Figure 4:**
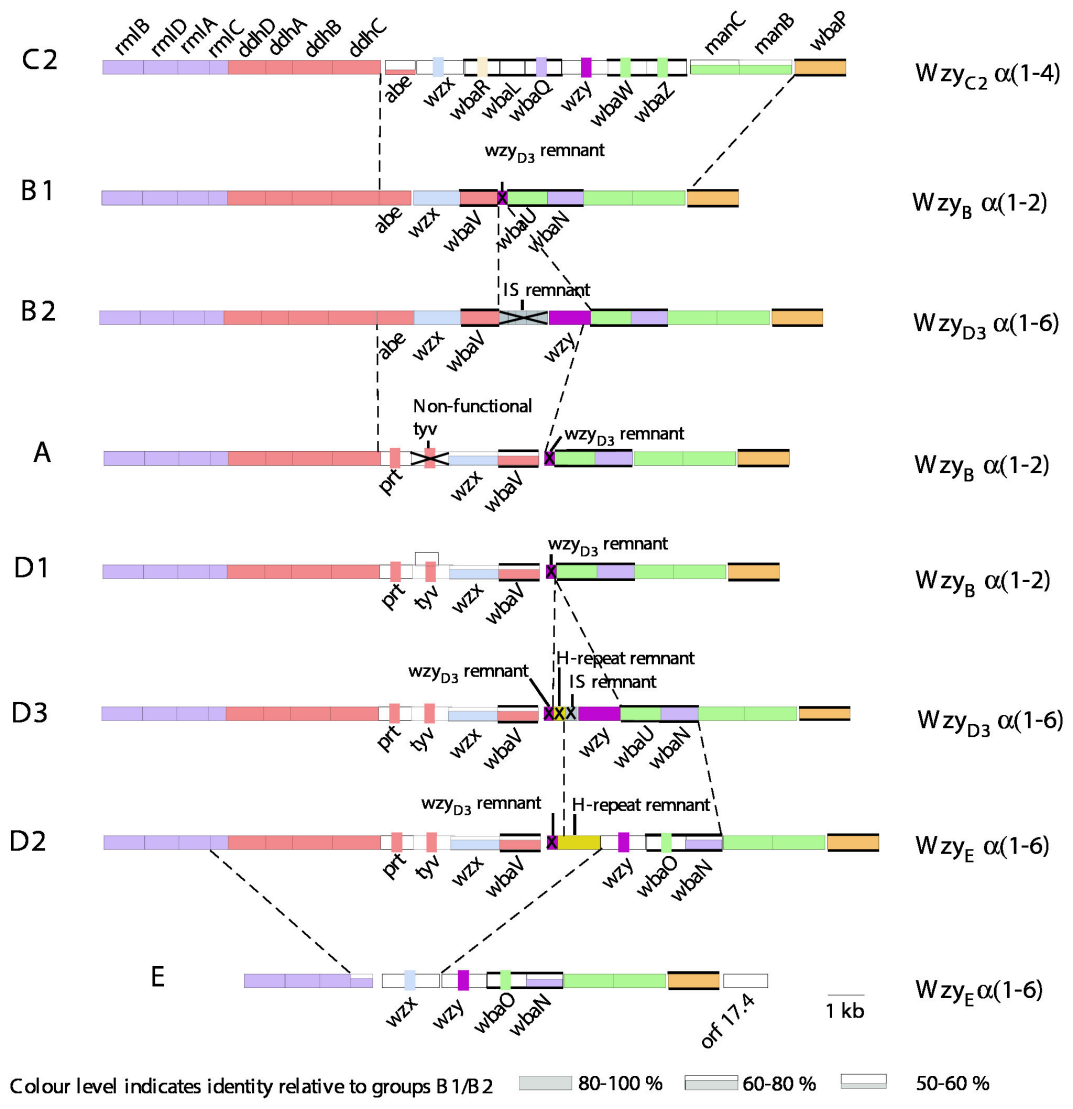
O-antigen gene clusters of the Galactose-initiated *S. enterica* serogoups. Genes are colour coded by synthesis pathways. Height of the coloured blocks indicates level of sequence similarity to group B1 genes; if little or no sequence similarity, the gene is colour coded by a vertical strip according to the pathway. Transferase genes are indicated by bold horizontal boundaries. Major junctions between blocks of genes with different relationships or levels of similarity are indicated by dashed lines. Gene names are shown and gene remnants labelled. The O-unit polymerase is shown on the right, named after the group in which it was first found, together with the linkage that it forms. Drawn to scale.

Groups A, B1, B2, C2-C3, D1, D2, D3 and E each have a distinctive gene cluster (Figure 4) with substantial overlap in genes present. After the strain LT2 B1 O-antigen gene cluster was sequenced [13], those of other groups were studied by first using restriction enzyme mapping to locate common regions, and then sequencing only regions that had not been sequenced previously [30–34]. We have resequenced these gene clusters to obtain full gene-cluster sequences for each group, and in some cases have additional sequence data from public genome sequences. The conclusions in this paper are based on full gene-cluster sequences, but note that some of the publications cited relied on sequence of the serovar specific regions only.

The functions have been determined for all biosynthetic pathway genes in the eight groups, and also those for the GTs [20,21]. The *wzy* and *wzx* genes have been identified for the 8 gene clusters but functional analysis has only been carried out on the group B1 Wzy polymerase [25], and Wzx flippase [23,24,35]. The chain length determinant gene, *wzz*, is about 3kb downstream of the O-antigen gene cluster, but is conserved and not discussed in this paper. Suffice to say that to our knowledge there are no reports of any functional variation in *wzz* in *S. enterica.*

With a few exceptions, each gene cluster includes all of the genes required for synthesis and assembly of the relevant structure starting from compounds generally present in *S. enterica*. The major exception of *wzy* in some groups is discussed below, while other genes involved in synthesis are also involved in other cellular housekeeping functions and map elsewhere: for example, *manA*, which codes for the first step in GDP-mannose synthesis, is also used for mannose catabolism [36].

Many genes are shared between two or more groups and in some cases, such as *rmlABCD*, by all groups. There is a modular structure for the gene clusters with, for example, the genes for synthesis of each NDP-sugar being found in a separate conserved module in a conserved location. The *rml* genes are at the 5’ end of each of the eight gene clusters, followed in all except group E by the four *ddh* genes, and then by the gene(s) required to complete synthesis of the relevant dideoxyhexose (DDH) sugar. At the other end of the gene clusters are the *manC, manB* and *wbaP* genes. The more diverse GT and processing genes are in the central regions, with each gene cluster having a specific set of GT genes and one each of the *wzx* and *wzy* genes. Thus for each pairwise comparison, all major sequence differences are in centrally located blocks, with the exception of wbaK in group E, a gene that is not uniformly present in all isolates of this group that is discussed below.

It is also apparent that while the genes for synthesis of each NDP-sugar precursor are clustered, they are not present in function order, which is indicated by the alphabetical order of the fourth character of the gene name. For example, *rmlABCD* is the function order but in the eight gene clusters the map order is *rmlBDAC*. Also the GT genes are not linked to the pathway genes for the associated NDP-sugar precursors, but are located within the central group of genes. Remarkably in each gene cluster the GT genes are transcribed in inverse order to their function in O-unit assembly [20], as can be seen by comparing the GT genes for each linkage (Figure 1) with the locations of the genes in the gene clusters (Figure 4). An apparent exception is *wbaP* that is not in the central block of genes, but is the last gene transcribed. However, it is the only transferase gene common to all gene clusters, so its presence at the 3’ end, instead of in the central block, is consistent with the general pattern for shared genes, and it does fit the pattern for the transcription order of GT genes as it encodes the initial sugar phosphate transferase.

### Groups B1 and B2 have an abequose side branch

The current group B is distinguished from other serogroups by having an abequose side branch on the Gal residue of the 3-sugar main chain found in 6 of the 8 groups. We propose that group B be divided into groups B1 and B2 as there are two structures based on differences in the location and sequence of the *wzy* polymerase genes and, that correspond to those in groups D1 and D3 respectively (Figure 1). The differences due to differences in the gene clusters (Figure 4), and the proposal fits the guidelines used for defining groups in *Salmonella* . However this change has not been formally accepted although we understand that it is highly probable that it will be (Weill pers comm.)

The LT2 *wzy*_B_ gene of the original group B was found outside of the main O-antigen locus, at a locus originally named *rfc*, but most other O-antigen gene clusters do contain a *wzy* gene. The new group B2 comprises the long-standing group B variant with the O27 epitope, that is due to presence of the α(1–6) polymerization linkage instead of the usual α(1,2) linkage. This α(1–6) polymerization linkage of the O27+ strains had been “shown” [37,38] to be conferred by a bacteriophage and therefore was not expected to be in the gene cluster. However when an α(1–6) *wzy* gene was found in the group D3 O‑antigen gene cluster, we found to our surprise that the *wzy*_D3_ gene is present in the gene clusters of all O27+ (B2) serovars, and a remnant is present in all O27- (B1) serovars. This α(1–6) *wzy* gene is between *wbaV* and *wbaU* in the gene cluster [39], and named *wzy*_D3_ as it was first found in group D3. It now appears that a converting phage was generated during the experiment in which a phage carrying the *wzy* gene was demonstrated [39].

Because the distinction has not been formally recognized it is not yet recorded how many B1 and B2 serovars exist, but the total will be more than the current 148, as there are H1/H2 combinations that occur in both B1 and B2.

The finding of the B2 gene cluster clarified the origins of the B1 gene cluster, which can now be seen to have a remnant of the B2 α(1–6) *wzy*_D3_ gene [39]. Clearly B1 arose from B2 by gain of a new α(1,2) *wzy* gene outside of the main gene cluster, and subsequent loss of function in the original α(1–6) *wzy* gene.

**Figure 5:**
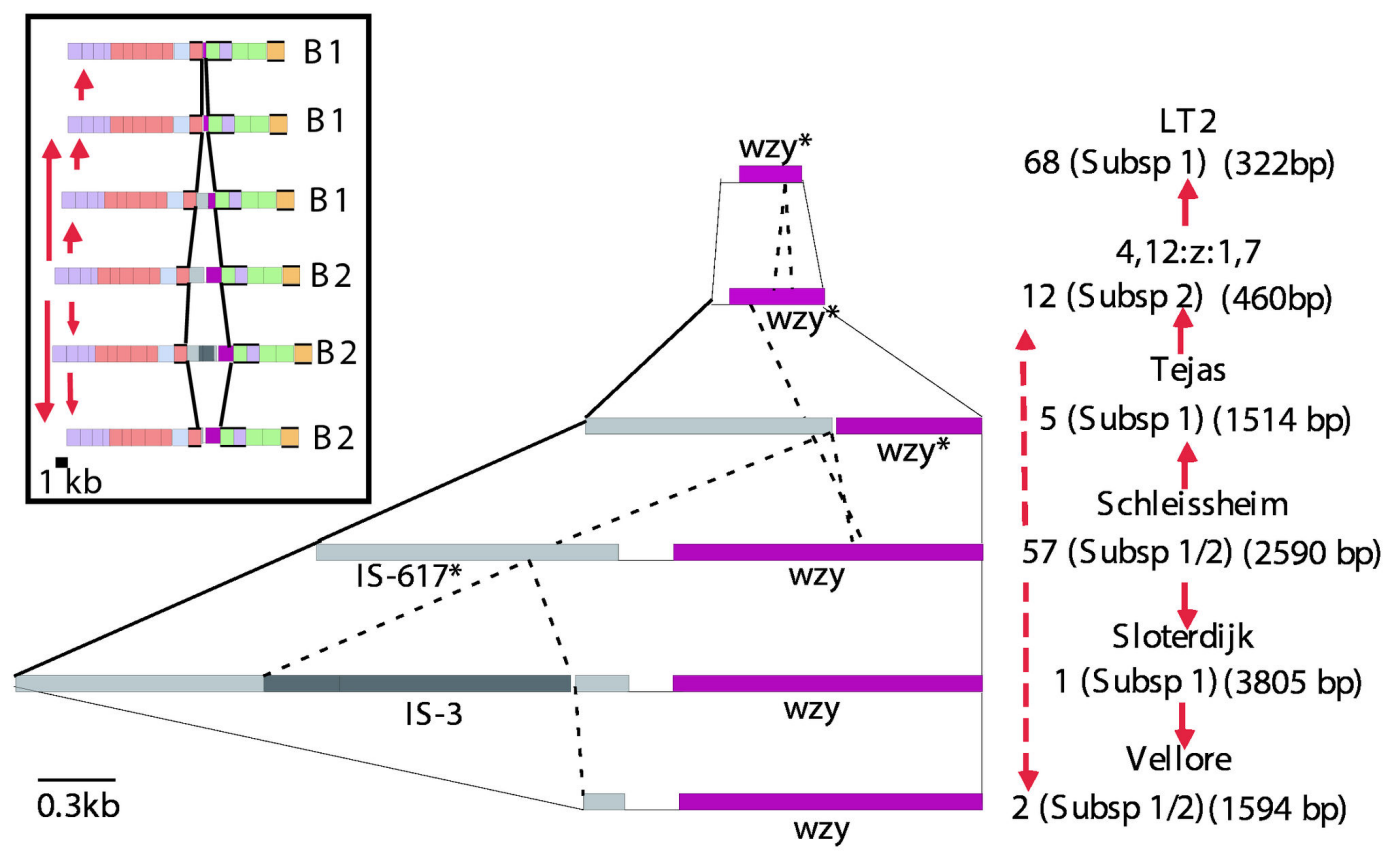
The 6 known group B1 and B2 forms of the region between *wbaV* and *wbaU.* This is an expansion of a small segment of Figure 4 to show detail of the differences. The box insert has a cartoon of the clusters as shown in Figure 4. Forms differ in the presence or absence of insertion sequences, and either a non-functional *wzy* remnant (marked with an asterisk; characteristic of B1), or the complete *wzy* gene that characterises B2. IS types are colour-coded and labelled. Ends of sequence deletion/insertions are indicated by dashed lines, and the junctions with the neighbouring *wbaV* and *wbaU* genes are indicated by solid lines. A representative of each form is named on the right, as is information regarding segment length, the number of isolates within each form and their subspecies distribution. The proposed diversification from an ancestral Schleissheim form to other forms is shown by red arrows. Dashed arrow lines indicate alternative pathways. Modified from [39]..

Six variants of the gene cluster were detected by variation in size of PCR products of the *wzy* region [39]. One of the sequence forms has what appears to be the ancestral B2 gene cluster, with the *wzy*_D3_ α(1–6) gene, and upstream of it a defective, but still large, IS1617-like element. We refer to it here as the Schleissheim form with 57 serovars (Figure 5). The other sequence forms are clearly derived from the Schleisshiem form by deletion or by insertion of another IS element, and it was possible to infer their relationships as shown in Figure 5. The Sloterdijk form (1 serovar) has gained an IS3 insertion and the Vellor form (2 serovars) has suffered a major deletion; both were O27+. The Vellor form lacks the IS3 sequence, so could have been derived by deletion from either the ancestral Schleissheim or the Sloterdijk form. In contrast, 12 subspecies 2. group B1 O27- strains of serovar 4,12: z: 1,7 have suffered a major deletion covering much of the *wzy* gene and all of the IS. Five other subspecies 1 serovars (the Tejas form) have a smaller deletion of *wzy* and the IS, that is within the larger region missing in the Sv 4,12: z: 1,7 form. This deletion in the 4,12: z: 1,7 form could have been derived directly from the ancestral Schleissheim form or sequentially via the Tejas form. However, most (68) subspecies 1 O27- serovars have the LT2 form and are clearly derived from the Sv 4,12: z: 1,7 form, as the LT2 form has the same major deletion plus a small additional deletion in a different part of the *wzy* gene.

The presence of the IS1617-like element in the ancestral Schleissheim form may itself indicate an earlier restructure in the region, but we have no putative ancestor for it. However, the variation in this IS sequence enabled us to study the relationships of the group B1 and B2 gene clusters. Note that most group B1 strains have a functional *wzy* gene at the old *rfc* locus well away from the main O-antigen gene cluster, but four of the serovars tested lack both *wzy* genes. They all have long-chain O antigen and must have another *wzy* gene elsewhere that is yet to be identified [39].

### Groups D1, D2 and D3 have a tyvelose side branch

The D1, D2 and D3 O antigens differ from those of B1 and B2 in having tyvelose in place of abequose as the side-branch sugar (Figure 1). Both sugars are immunodominant and this was the basis of the early differentiation of groups B and D by serology, with epitope 4 due to abequose defining group B, and epitope 9 due to tyvelose defining the original group D. The differences between D1, D2 and D3 lie in the mannose-rhamnose (Man-Rha) linkage in the repeat-unit backbone and the polymerization linkage (see Figure 1). The D1 gene cluster closely resembles that of group B1 with only four genes differing significantly (Figure 4). Most of the B1 and D1 sequences are near identical, but following the shared *ddh* genes, D1 has *prt* and *tyv* in place of the *abe* gene of B1, relating to DDH specificity (see Figure 3), and the adjacent *wzx* and *wbaV* genes also differ substantially in sequence. There are well-demarcated junctions between the near-identical genes and those in the central divergent region. WbaV is the transferase for abequose or tyvelose, in B and D1 respectively, and WbaV, Wzx and Wzy are the only proteins that act after addition of the DDH side-branch sugar to the O unit, giving them different substrates in B1 and D1. The *wzy* gene is at the *rfc* locus and is the same gene in both groups, but it is possible that the sequence divergence in *wzx* and *wbaV* reflects differences in substrate specificity. It is known from *in vitro* studies with a group B1 membrane preparation, that incorporation of tyvelose is only 20% that of abequose under the same conditions [40] suggesting that the B1 WbaV GT is more efficient with its normal substrate. It appears that some of the sequence divergence could be due to the substrate difference, but there is no data on the D variant of WbaV. However the WbaV GTs of both B1 and D1 strains were shown to incorporate both sugars by complementation of *abe* or *prt*-*tyv* mutations in each with the alternate gene(s) [41], although this is not a quantitative test.

Group D1 resembles B1 in having the α(1,2) polymerisation linkage, with the same *wzy* gene at the *rfc* locus. Other *S. enterica* genomes (listed in Table 1), from groups B2 (1), A (1), D1 (5) and C2 (5), were screened for the *rfc*-locus *wzy* gene of LT2 using the ARTEMIS Comparison Tool (http://www.webact.org/WebACT/home [42]. This indicated that it is present in groups A and D1, but not in groups B2 or C2 (data not shown), which correlates with the presence or absence of the α(1,2) polymerization linkage.

The group D1 gene cluster also contains a *wzy* remnant between *wbaV* and *wbaU* as in group B1 [31], but it is shorter than in B1, and also covers a different region of the original gene suggesting a different pathway of gene degradation.

### Group D2 arose by recombination between groups D1 and E

**Figure 6:**
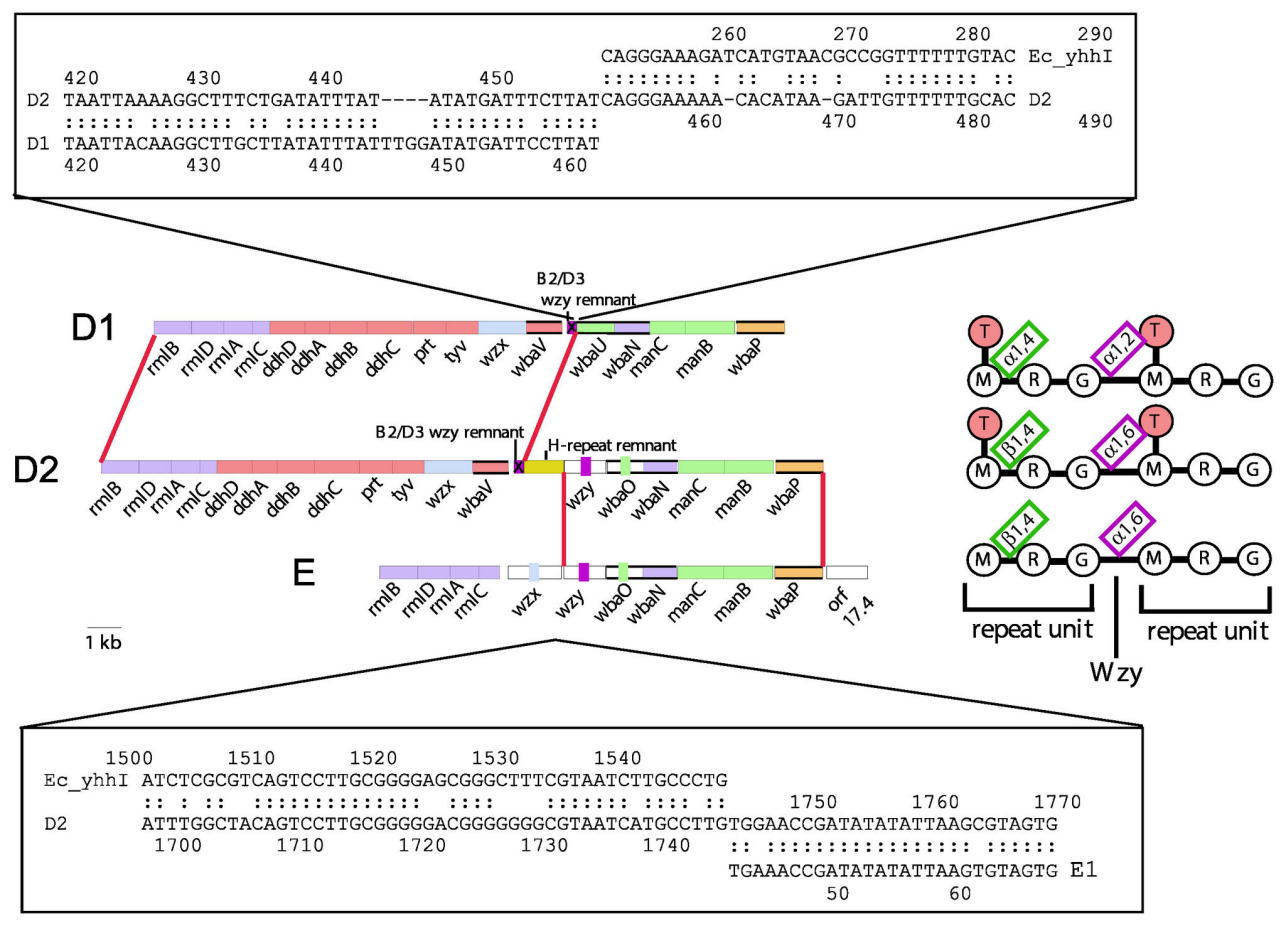
Relationships of group D1, D2 and E gene clusters. Gene clusters of groups D1, D2 and E are shown, with the D2 regions proposed to be derived from D1 and E indicated by red connecting lines. Genes are colour-coded as in other figures. Sequence alignments of D2 with D1 (above) and with E (below) are shown for the junctions between homologous and non-homologous sequence, with the *E. coli* K-12 H-repeat element, *yhhI*, used for the H-repeat comparison. Cartoons of 2 O-unit repeat structures of each group are shown to the right to highlight the structural differences that relate to the genetic differences between clusters: purple, polymerisation linkage; green, Man-Rha glycosidic linkage; orange, DDH sidebranch. Abbreviations: G, galactose; M, mannose; R, rhamnose; T, tyvelose.

**Figure 7:**
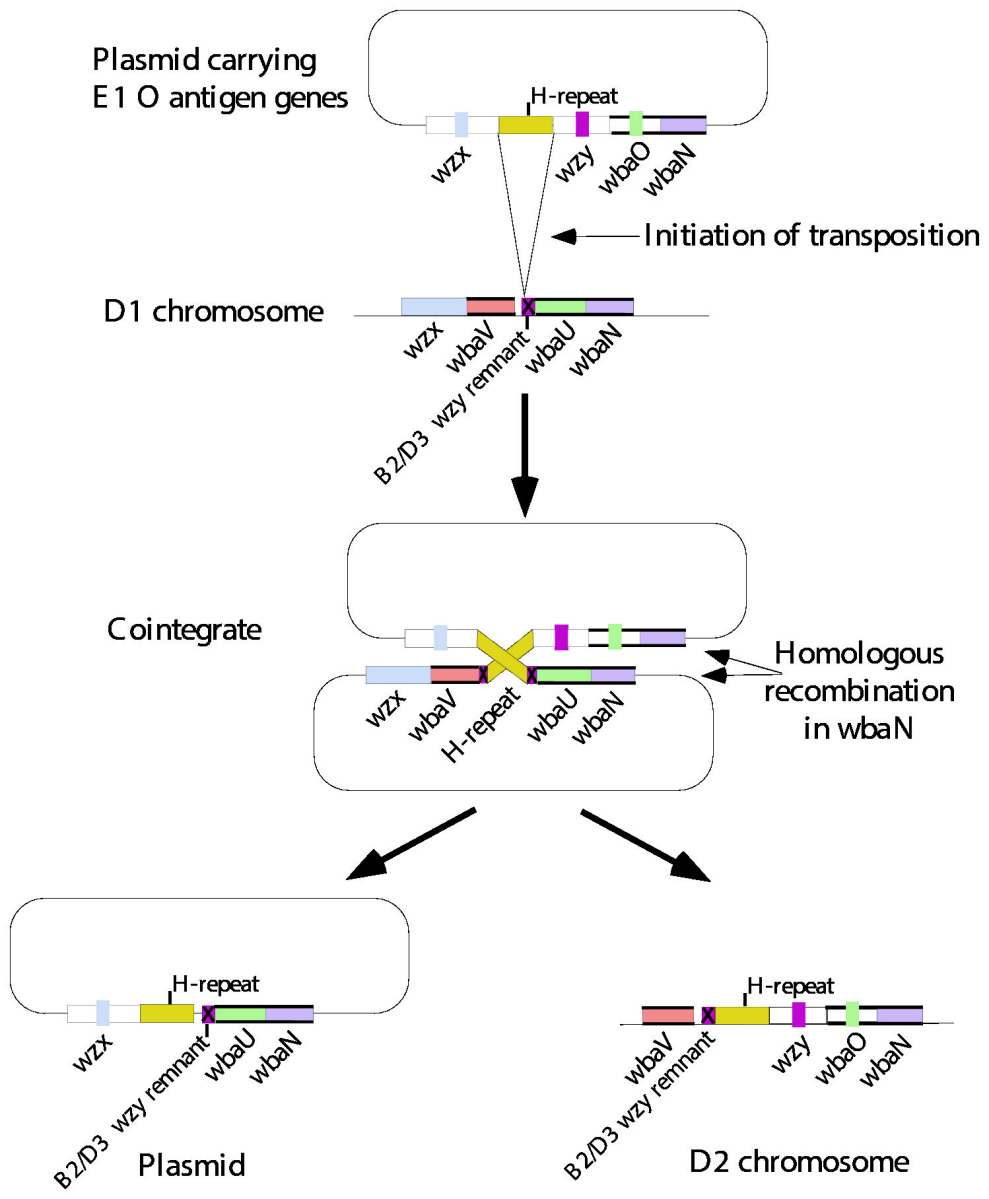
Model for the origin of the D2 gene cluster by “recombination” between groups E and D1 gene clusters. An H-repeat element present on a plasmid-based E-like gene cluster mediates a transposition event into the D1 gene cluster, with “resolution” of the intermediate cointegrate by homologous recombination in *wbaN*, instead of by a resolvase. Model based on replicative transposition. Modified from [32]..

Group D2 has an α(1–6) Gal-Man polymerization linkage, instead of the α(1,2) linkage in D1 and B1 strains, and also has a β(1-4) Man-Rha linkage within the repeat unit instead of the α(1–4) linkage of D1 and B1 (Figure 1). In both cases, D2 resembles subgroup E1, which we discuss below, but has the tyvelose side branch of D1. Comparison of the gene clusters suggested that D2 has arisen by recombination of group D1 and E gene clusters [32], as D2 has the 5' end of the D1 cluster, including the DDH-pathway genes, and the 3' end of the E cluster including the β(1-4) Man-Rha GT gene and the *wzy* gene [32] (Figure 6). There is a remnant H-repeat element at the junction, which was proposed to have been involved in the recombination event to give rise to D2 (Figure 7) [32].

### Group D3 appears to be the ancestral D form

D3 is very unusual in that it has two repeat-unit forms: one with an α(1–4) Man-Rha linkage, and the other with a β(1-4) linkage. The D3 gene cluster [31] has the *wbaU* gene of D1, which accounts for the α(1–4) Man-Rha linkage, but no additional GT for the β(1-4) form and we assume that this maps elsewhere. Both D2 and E1 gene clusters include a *wbaO* gene responsible for the β(1-4) Man-Rha linkage, but PCR screening with *wbaO*-specific primers in D3 did not reveal the presence of this gene in the D3 genome [31].

D3 has an α(1–6) polymerization linkage between repeat units, and the same *wzy* gene is present within the gene cluster as in B2; indeed it was first found in D3 [31] as discussed above. This *wzy* gene, however, is different to that found in D2 and E, which also encodes an α(1–6) polymerase.

**Figure 8:**
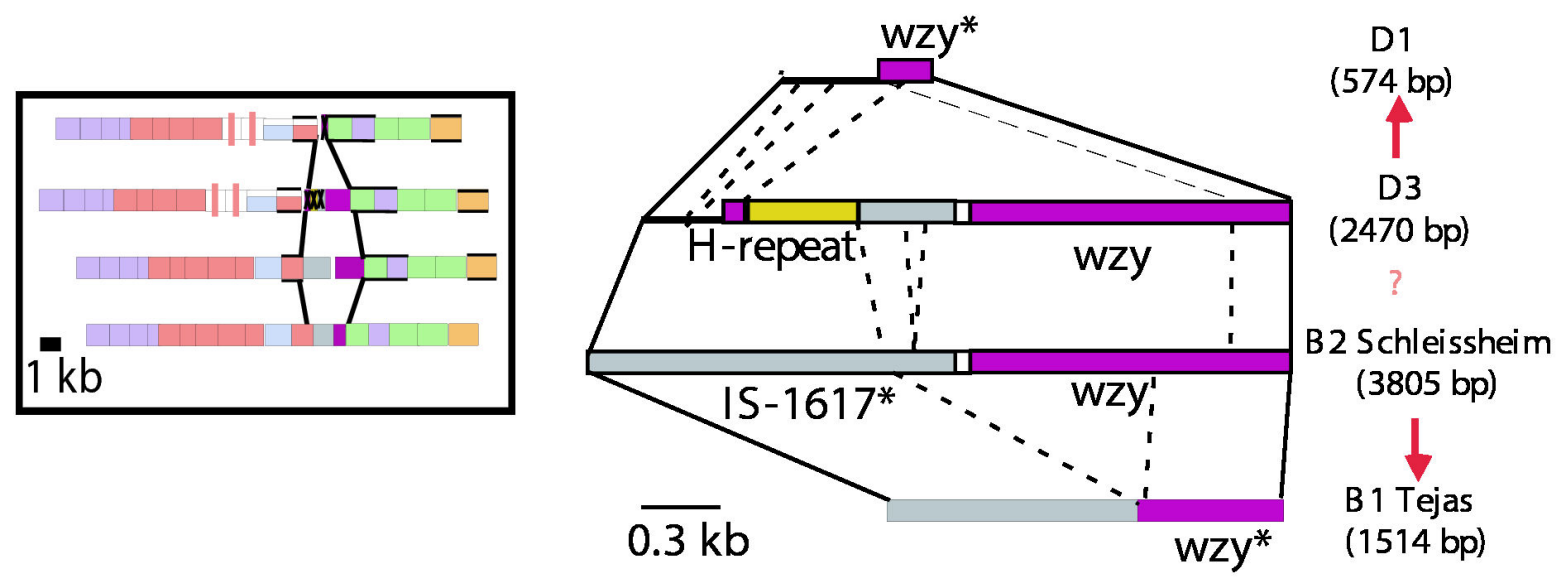
Comparison of the segments between the *wbaV* and *wbaU* genes of Groups D1, D3, B2 and B1. Base positions are indicated and are taken from either published or deposited sequences, as indicated in Table 1. The junctions with the neighbouring *wbaV* and *wbaU* genes are indicated by solid lines. A box cartoon to the left shows the gene clusters from Figure 4. Red arrows indicate a proposed diversification pathway of segments.

It is interesting that the relationship of *wzy* regions of D3 and D1 is the same as that for B2 and B1 [31], suggesting that D1 arose from D3 in the same way as proposed above for B1 and B2 respectively. In Figure 8, an analysis of the *wbaV-wbaU* intergenic region is depicted for the group D1 and D3 sequences, and their relationship to each other, and also to groups B1 and B2. Analysis of the derivation of D1 from D3 is more difficult than for B1 from B2, as the D3 sequence has a duplication of part of the *wzy* gene (Figure 8) that probably occurred after the deletions(s) that gave rise to the D1 form. The IS-fragment present in D3 is from same IS1617-like sequence that is present in B2, and the *wzy* remnants in D1 and B1 are from the same *wzy* gene, but differ as they have undergone different pathways for degradation by serial deletions. However, it is clear that groups D1 and D3 share the 5’ end of the gene clusters, and also part of the intergenic region after *wbaV*, but the sequences then diverge further downstream (Figures 4 and 8). The implications of these observations are discussed further in the section on gene cluster relationships.

### Group A is a single-gene mutant form of Group D1 with a paratose side branch

**Figure 9:**
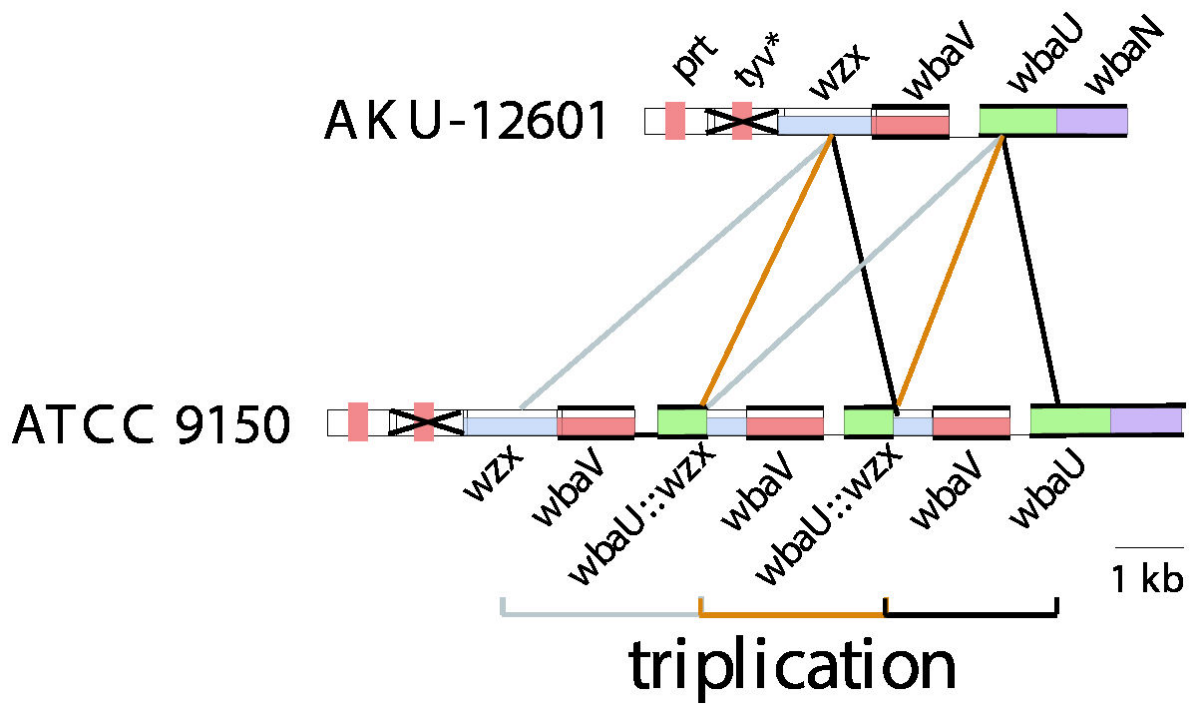
Group A triplication. Alignment of group A sequences with and without the triplication of the *wbaV* region. Strain names are indicated on the left (see Table 1 for details). Both strains have identical gene clusters with the exception of the triplication of the region indicated. Drawn to scale.

The gene cluster of the serovar Paratyphi A group A strain initially studied was found to contain 2 significant differences from D1 gene cluster. The first was a frame shift mutation that generated a stop codon reducing the CDP-tyvelose epimerase to 5 residues, which would prevent conversion of CDP-Paratose to CDP-Tyvelose (Figure 3), and accounts for the presence of paratose in place of tyvelose, the only structural difference between the A and D1 O antigens. The other difference was a triplication of one segment, which led to an increased copy number of the *wbaV* gene, and generated a *wbaU::wzx* chimeric gene at the recombination site for the triplication [33] as shown in Figure 9.

Nine other Paratyphi A strains were studied using PCR and all but one had a replication of the *wbaV* region, with from 2 to 4 copies of the repeated segment [41]. Also both Paratyphi A genome sequences have the same frame shift mutation in the *tyv* gene, but one has the triplication event and the other not. Other than this triplication, the two cluster sequences are identical. It should be noted that replications of this type are not uncommon and the number of replicates can expand or contract during chromosome replication by slipped strand mispairing [43], but once it returns to zero then it cannot expand back by this process. Single strains of serovars Nitra and Kiel were studied by PCR and neither gene cluster had the replication event [41]. The remaining group A serovar, Koessen, had not been reported at the time of those experiments, and there is no information on its *wbaV* region.

It appears that group A strains have arisen from D1, and probably very recently, as there are only 4 group A serovars reported in the 2007 KW scheme, and the *tyv* gene, although non-functional, is still present in the three group A serovars available at the time [41]. The *tyv* gene is not required in group A, and its presence indicates that the group A forms arose from group D by loss of *tyv* function. This is in contrast to the situation in *Yersinia pseudotuberculosis* in which serovars with paratose have the *prt* gene, but no *tyv* gene or remnant [44].

### Group E

Group E has several distinguishing features. It differs from all other Gal-initiated groups in not having a DDH, and in relation to the several B and D groups, other than D2 (as discussed above), in having a β(1-4) Man-Rha linkage instead of an α(1–4) linkage. The gene cluster lacks any evidence of ever having had the DDH-pathway genes. There is an intergenic gap between the last *rml* gene and *wzx*, where these *ddh* genes occur in the others, but we find no remnants of *ddh* genes. The β(1-4) Man-Rha linkage is made by GT WbaO, encoded by a gene that replaces the *wbaU* gene for the α(1–4) linkage of B1, B2, D1 and D3. Group E also has an α(1–6) *wzy*_E_ gene in the central region that, as discussed above, is not related to that of B2 and D3, but is thought to be the source of the *wzy* gene of D2. Finally, group E has a very different *wzx* gene to the related pair of *wzx* genes found in groups B1 and B2, and in D1, D2 and D3 respectively, which have different DDH side-branches.

**Figure 10:**
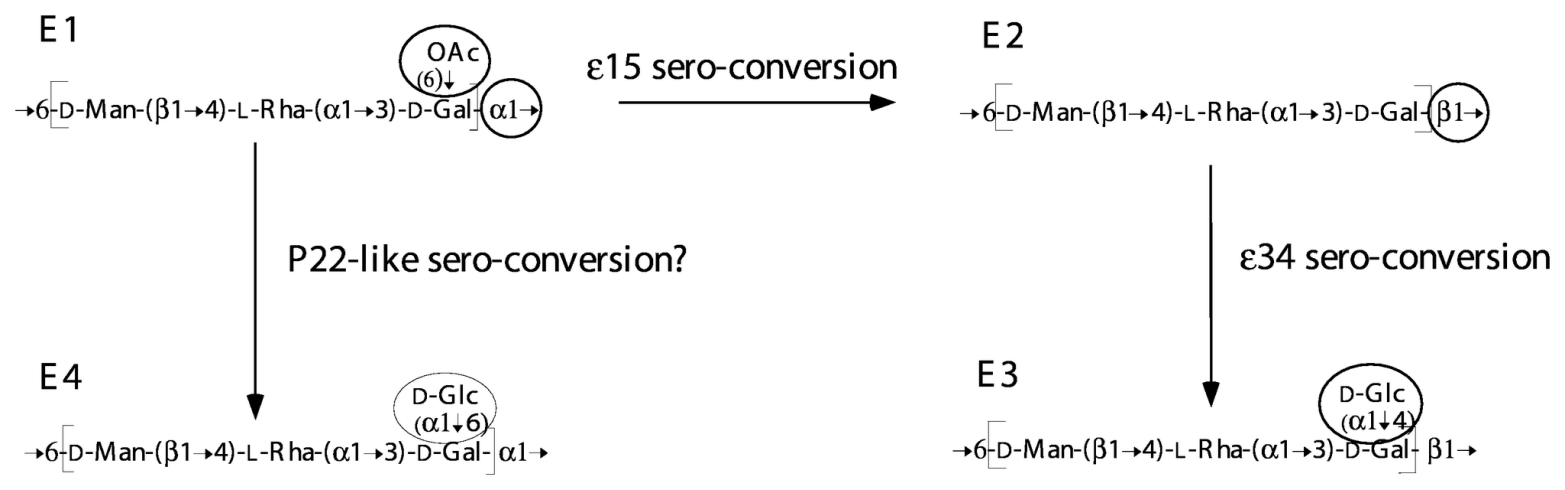
O-unit serogroup modification in Group E. The group EO unit is modified by phage genes that result in either glucosylation, O-acetylation, or a different O-unit polymerisation linkage being produced. The moieties undergoing change are circled and the differences define the 4 known group E structural variants: E1, E2, E3 and E4. The e15 and e34 phage are well known and modification to form an E4 variant is thought to be carried out by a P22-like phage (see text for details).

Group E was initially subdivided into groups E1, E2, E3 and E4, based on serology, and the structures are all known (Figure 10). The genes for the three-sugar main chain, shared by all four of the earlier groups, are in the O-antigen gene cluster. The E2 and E3 variants of the E1 structure are due to presence of bacteriophages, and these are no longer treated as groups but have been rolled into group E1 in the current (2007) KW scheme. We sequenced an E4 gene cluster and it also has the same gene cluster, and so all should be treated as being in a single group E (Weill pers comm.). Indeed the additional glucose (Glc) residue in the E4 form was shown quite early to be due to an *oafC* locus close to *purE* [45].

The e15 and e34 bacteriophages are responsible for the O15 and O34 epitopes of E2 and E3, respectively. The e15 phage has three properties: suppression of the O-acetyl transferase activity, synthesis of a β(1–6) polymerase, and suppression of the α(1–6) polymerase [46–48] [49]. The phage e15 genome [50] includes a *wzy* gene, a gene for an inhibitor of the original Wzy protein, and two genes for short proteins that inhibit O-acetylation of the Gal residue. This is a remarkably complex suite of genes for a serovar conversion event. Phage e34 confers a glucosyl transferase property that is only expressed in the presence of the beta polymerization linkage conferred by e15, presumably due to specificity of this transferase. The e34 genome has been sequenced and shown to have a *gtr* set of genes that resembles those found for addition of other side-branch Glc residues, and is proposed to be responsible for this function [51]. The detail of the *oafC* locus in E4 strains is not known, but also probably involves a *gtr* gene set.

There is a complication in relation to the β(1–6) polymerase *wzy* gene in this group. The group E1 gene cluster sequenced first had an additional gene, *wbaK*, after *wbaP*, originally named *orf*17.4 [52]. At the time it was predicted to be a second *wzy* gene within the cluster, but it now seems clear that WbaK is the O-acetyl transferase for the O-acetyl residue found in subgroup E1 (unpublished data).

### Group C2-C3 has a unique backbone structure but the same sugars as group B

Group C2-C3 has the same sugars as group B, but the order differs, and there are no shared linkages (Figure 1). This is reflected in the gene cluster (Figure 4), in which the rhamnose, DDH and mannose biosynthesis genes are present in the same arrangement as in the other groups, but all of the genes in the central region are unique to C2-C3, except for *abe* at the 5’ end of this region which is 45% identical to the group B gene at the amino acid level [21]. Interestingly, the gene order in the central region follows the same pattern as in other groups, with the *abe*, *wzx* and dideoxyhexose transferase (*wbaR*) genes first, followed by the other GT genes in the reverse order to their function. Even the *wbaL* O-acetyl transferase gene maps according to its function order, as the O-acetyl residue is added to rhamnose prior to addition of the abequose residue by WbaR [21]. All expected functions are accounted for.

**Figure 11:**
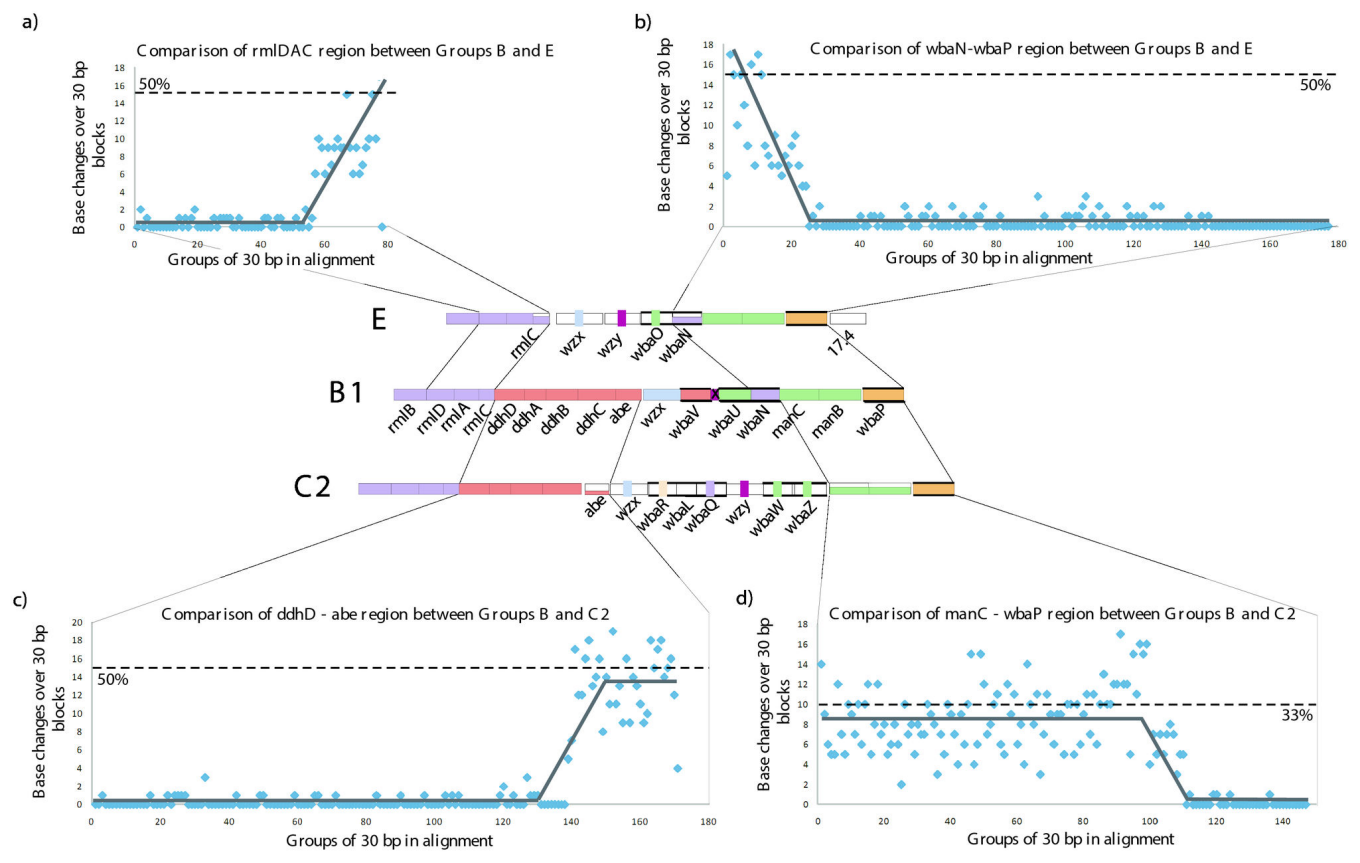
Sequences comparisons for the junctions in alignments of group B with group E or group C2-C3 gene clusters. Pairwise alignments of nucleotide sequences were used to generate plots. Base differences or gaps were given a value of 1, identical bases a value of 0. Divergence was plotted using the values for 30 bp non-overlapping segments for each alignment. a) groups B1 and C2-C3 *ddhD-abe* segments; b) groups B1 and C2-C3 *manC-wbaP* segments; c). groups B1 and E *rmlADC* genes; d) groups B1 and E *wbaN-wbaP* segments. Divergence trends are indicated with solid lines. Gene clusters for groups B, C2-C3 and E are shown in the centre for reference.

C2-C3 is unusual among the eight groups studied here in that there are no reported gene cluster encoded variants, like those seen for B1 and B2; D1 and A; D1, D2 and D3. However, this group was divided, like group E, into subgroups (C2 and C3) which have the same O-antigen gene cluster, but C3 has a Glc sidebranch on the Gal residue that does not seem to have been studied in detail and is presumably encoded by a *gtr* set of genes elsewhere on the chromosome. Comparison of the junctions between genes in groups C2-C3 and B1 (Figure 11a and 11b) demonstrates similar patterns to those seen for some other groups, with regions of high levels of divergence present in the shared genes adjacent to the central differing block of genes in the two clusters. Possible reasons for this and the implications are discussed in the next section.

### Relationships of the eight Gal-initiated gene clusters

Of the eight O antigens studied, all but C2-C3 have closely related structures and gene clusters, and whenever two genes carry out parallel steps during biosynthesis, they are at the same location in the gene cluster. This applies to *wbaU* and *wbaO* for α(1–4) and β(1-4) Man-Rha linkages respectively, and to *abe* and the *prt*-*tyv* pair of genes, which code for the final steps in biosynthesis of Abe and Tyv. It also applies to the different *wzx* and *wzy* genes (including *wzy* remnants) found in the seven groups. However, the gene clusters have several levels of divergence for homologous genes, as shown in Figure 4, and we need to consider the levels of sequence divergence in context. In some cases the divergence is within the range found for house-keeping genes within a species, but in other is much greater and we will distinguish three levels of divergence.

### Divergence at within-species levels – movement of O-antigen gene clusters between subspecies

The lowest level of divergence is seen in the *rml*, *man* and *ddh* gene sets, and in *wbaP*, These genes are in all or most of the gene clusters for the Gal-initiated O antigens and divergence is often within the range for housekeeping genes. The great majority of *S. enterica* isolates are from these serogroups, so we can in effect treat the genes as housekeeping genes, because the genes are at the ends of the gene cluster, and the gene cluster boundary will have little or no effect on the random genetic drift that limits accumulation of mutations, and maintains the low level of sequence variation in housekeeping genes. A significant number of the WecA-initiated gene clusters also have the *rml* genes at the 5’ end and so for these genes there is even greater opportunity for random genetic drift.

*S. enterica* has relatively little variation within subspecies compared with variation between subspecies, and this correlates with high recombination within, but not between subspecies [4,53,54]. The 2007 KW listing shows that many O-antigen forms are found in more than one subspecies, which implies either transfer of the gene cluster via recombination between subspecies, or inheritance from the common ancestor of the subspecies involved. If recombination then the ends of the DNA segments proposed to have been transferred by homologous recombination could have been within the common genes that flank the gene clusters, or within those parts of the gene cluster that are shared as discussed above. There is in fact good evidence for recombination occurring within the *rml* gene sets from a study on 35 strains representing 13 *S. enterica* serogroups that have rhamnose in their structure [55]. For example, variation in *rmlC*, the gene adjacent to the central O-antigen–specific region, is generally correlated with the O-antigen form, while variation in *rmlB*, at the start of the gene cluster, is often correlated with subspecies. The overall pattern was consistent with *rml* genes diverging with the subspecies and occasional transfer of the gene cluster between subspecies involving a recombination junction within one of the *rml* genes.

A comparison of the B1, B2, D1 and D3 gene clusters is particularly interesting in the light of subspecies variation. The D3 gene cluster is from a subspecies 2 isolate, whereas the gene cluster sequences available for the other O antigens are all from subspecies 1 isolates. For genes shared by all 4 gene clusters, the D3 genes differ from the others by 1.4 to 6.2%, whereas the variation in the shared genes of the subspecies 1 gene clusters ranges from 0-1.4% (Table 2), except for those cases to be discussed below where divergence is outside the range for intraspecies variation (see Table 2). The range of 1.4 to 6.2% is consistent with divergence between subspecies 1 and 2 for housekeeping genes.

**Table 2:** Levels of within-subspecies and between-subspecies identity for shared genes.

Pairwise Gene Comparison	Subsp I/ Subsp I	Subsp I/ Subsp II	Subsp I/ Subsp II	Subsp I/ Subsp I	Subsp I B27/ Subsp II-D3	Subsp I/ Subsp I	Subsp I Subsp I	Subsp I/ SubspII
*Gene*	D1/D2	D2/D3	D1/D3	B27/B	B27/D3	B/D1	B/D2	B/D3
*rmlB*	99.5	95.7	95.6	99	95.8	99.5	99.6	95.7
*rmlD*	98.6	94.9	94.7	97.9	95.1	98.6	98.1	94.1
*rmlA*	98.6	93.8	94.1	98.6	93.8	98.6	98	94
*rmlC*	99.8	96.5	96.7	98.4	97.4	99.1	98.9	96.5
*ddhD*	99.7	96.3	96	99.3	96.2	99.1	99.4	96.1
*ddhA*	99.9	98.4	97.9	99.9	98.2	99.6	99.5	98.3
*ddhB*	99.5	97.8	98.5	99	98.1	99.7	99.4	98.6
*ddhC*	100	97.5	97.5	99.5	97.6	99.4	99.4	97.6
*abe*	-	-	-	99	-	-	-	-
*prt*	100	95.6	95.6	-	-	-	-	-
*tyv*	99.8	96.9	97.1	-	-	-	-	-
*wzx*	98.7	93.9	94.6	98.3	-	65	62	64.8
*wbaV*	99.6	93.7	93.9	98.6		61.9	61.8	61.8
*wzy*	-	56.2	-	-	96.3	-	-	-
*wbaU*	-	-	96	98.5	96	99.8	-	96
*wbaN*	76.7	74.2	97	98.9	96.3	100	76.7	97
*manC*	99.7	96.4	96.4	98.7	96.3	99.9	99.6	96.2
*manB*	98.7	94.6	94.7	98.1	94.6	99.9	98.7	94.8
*wbaP*	96.4	95.9	96.4	99.5	96.5	99.9	98.8	96.4

If the sequence differences did arise during subspecies divergence, then the genes were in the subspecies’ common ancestor, implying that at least group D3 was in that common ancestor as the *prt*, *tyv* and *wbaV* genes were included in the D1/D3 comparison and had 4.4, 2.9 and 6.1% divergence. The B2 and D3 *wzy* genes had 3.9% divergence suggesting that B2 was also in that common ancestor. It appears that during subspecies divergence the genes shared by the D groups and the B groups evolved together by concerted evolution [56], to give the subspecies I and subspecies II forms that we see today.

The group D3 antigen has been found only in subspecies 2, but those of B2, B1 and D1 are found in both subspecies, and we need further data from both subspecies to confirm this hypothesis. It is particularly interesting that the *wzy* genes in B2 and D3 fit into this pattern, differing by 4%, which is consistent with this gene also being present in the ancestor of subspecies 1 and 2, and diverging as the subspecies diverged. At some stage the *wzy*_B_ gene entered both subspecies and the *wzy*_D3_ gene decayed to generate the B1 and D1 gene clusters. At present we have no information on the timing or order of the events.

### High-level sequence divergence predating Salmonella origins - evidence for divergence of groups long before the gene clusters reached *S. enterica*

#### Group E

The second level of divergence is much greater than normally found within a species. Our first example is group E, which lacks a DDH sugar and the *ddh* and associated genes. There is a central serogroup-specific set of genes from *wzx* to *wbaO* as discussed above (Figure 4) and outside of this all genes are shared with groups B1, B2, D1 and D3. Most of the genes are near identical in the four groups, but adjacent to the central region the group E sequence is substantially divergent. At the upstream junction the divergent region comprises the end of the *rmlA* gene plus all of *rmlC*, and downstream it covers just the *wbaN* gene (Figure 11c and 11d). These genes have the same functions in all groups, and divergence presumably reflects random genetic drift over a very long period. The divergence observed in these genes is not uniform but there is a gradient with divergence greatest at the boundary with the central gene blocks, suggesting that the sequence proximal to each form of the central block has remained with that block during this divergence, but that occasional recombination has reduced the level of divergence further from the junction, reaching near identity about 1kb from each junction. If this is the case, then divergence must have begun long before the origins of *Salmonella* as a separate genus, because the divergence is about 50% at both junctions in the group B1 and E sequence alignments.

If the hypothesis is correct then this places the divergence of group E from those with a DDH side branch to that distant period. It does not seem likely that this divergence is due to selection for sequence changes, as the substrates and products are the same for the 3 enzymes involved. However, for *wbaN* in particular, there could be selection for protein–protein interaction with other GTs, but this is very unlikely for the *rml* genes. Even if selection was operating, it would still take a substantial period of time for such levels of difference to accumulate. However, even without selection for differences, the situation observed in these regions could be quite stable once the divergence arose, because the level of difference would reduce recombination frequency. Thus while *rmlB*, *rmlD* and part of *rmlA* at one end of the gene cluster, and *wbaP*, *manB* and *manC* at the other end, could evolve in concert in all of these groups and remain as sites for recombination, the region that has now diverged significantly comprising the remainder of *rmlA*, all of *rmlC*, and *wbaN* of group E would continue to diverge from the genes in the other groups due to the recombination barrier created by the divergence itself. The gradient in the level of divergence discussed above would arise if occasional recombination events shortened the region of divergence, which then continued to grow slowly as before, but of course would never catch up with the original level of divergence. The existence of a gradient suggests that there were several such recombination events.

The extent of divergence at the junction with the central region is a measure of how far back the two central region sets of genes have been at this site, and reflects the minimum time for the origin of the two O antigens.

#### Group C2-C3

A similar situation occurs in C2-C3, with *manC*, *manB* and part of *wbaP*, that flank the 3’ end of the central serogroup-specific block, being very divergent (~30%) in relation to all other Gal-initiated groups, and at the 5’ end the *abe* genes are even more divergent (45%) in relation to groups B1 and B2, which also have the *abe* gene (see Figure 11a and 11b). At the 3’ end there is a substantial block of DNA with a consistent level of divergence adjacent to the junction followed by a gradient to the region with low-level divergence, but the region with consistent divergence is much shorter at the 5’ junction. It is interesting that the *abe* genes of groups B and C2-C3 are much more divergent (45%) than the *manB* and *manC* genes (~30%) at the 2 ends of the central block of genes that determine the differences between these groups. The situation is similar to that for group E discussed above, and the implication is that the C2-C3 gene cluster diverged from the others at least as long ago as the time required to attain these levels of sequence divergence. The reason for the difference in divergence levels at each end of the C2-C3 divergent segment is not known.

#### Groups B and D

**Figure 12:**
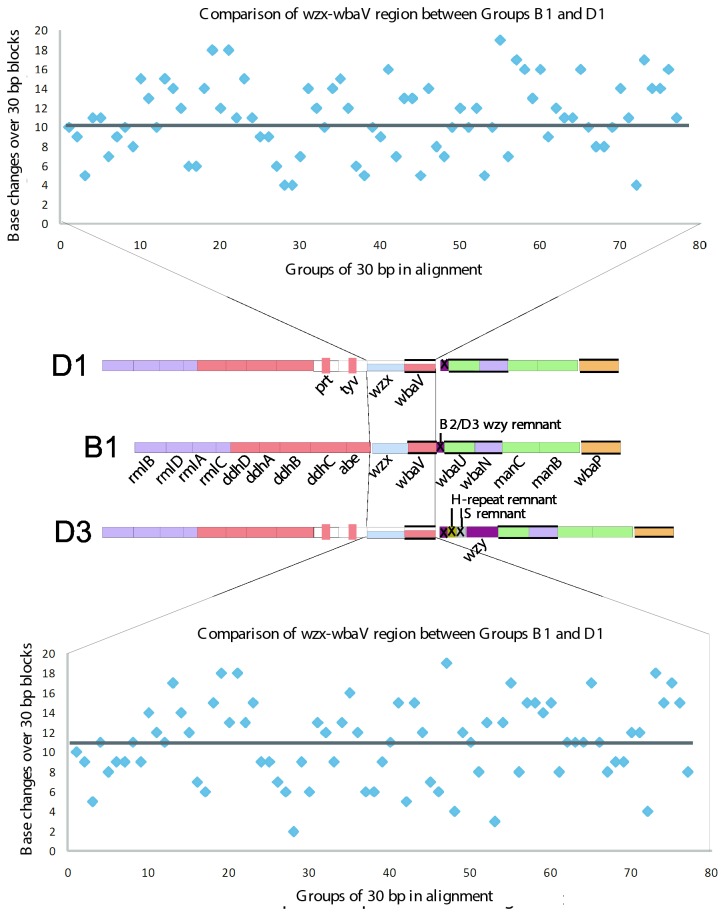
Sequence comparisons for the group B1, D1 and D3 *wzx-wbaV* gene blocks. Pairwise alignments of nucleotide sequences were used to generate plots. Base differences or gaps were given a value of 1, identical bases a value of 0. Divergence was plotted using these values over 30 bp non-overlapping bases of each alignment. Groups B1 and D1 are in the upper panel; groups B1 and D3 in the lower panel. Divergence trends are indicated with solid lines. Gene clusters for B1, D1 and D3 are shown for reference.

Another example is the relationship of groups B1 and B2 to groups D1 and D3. The *wzx*-*wbaV* blocks differ by about 33% (Figure 12). This pair of genes are adjacent to the serovar-specific genes of *abe* in B1 and B2 and *prt*-*tyv* in D1 and D3, which will generate the different side-branch sugars, thereby changing the O unit that will be flipped by the Wzx proteins, and the sugar to be transferred by WbaV. We know that either sequence form of WbaV can transfer abequose or tyvelose, or indeed paratose of group A, and for Wzx either form can flip the corresponding O units, as shown by complementation experiments (see above). However, this does not mean that these proteins act with equal efficiency on all substrates. In fact as discussed above, it has been shown that WbaV from LT2, with CDP-abequose as its normal substrate, transfers tyvelose from CDP at 20% the efficiency of abequose transfer, and it did not transfer paratose at a level detectable in the *in vitro* assay [40]. In this case the sequence differences could be due to selection relating to the difference in substrates, or to random genetic drift, but again indicates that the distinction between groups B1/B2 and D1/D3 is ancient.

### Extreme sequence divergence in homologues at the same locus

The third and highest level of divergence is found for genes that are related, with clear indications of homology, although the functions are different, for example the *abe* and *prt* genes of groups B and D that have partial identity of 56% at nucleotide level (Figure 4). In this particular case the substrate is the same, but the functions are different, with the keto group on carbon 4 being reduced to the alternative anomeric forms (Figure 3). These two genes, both found immediately after *ddhC*, may have diverged *in situ* from an ancestor to become functionally distinct.

### Diversity of Wzy and Wzx proteins

There are four different *wzy* genes present in the eight gene clusters discussed: *wzy*_C2_, *wzy*_E_ (also in D2), *wzy*_B_ at the old *rfc* locus (also in D1 and A), and *wzy*_D3_ (also in B2). These differences relate to the linkages involved. There are also four different *wzx* genes present in the eight gene clusters: *wzx*_C_ in group C2-C3, *wzx*_E_ in group E, *wzx*_B_ in groups B1 and B2, and the related *wzx*_D_ in groups A, D1, D2 and D3. The *wzx*_B_ and *wzx*_D_ genes have 65% nucleotide identity and have been discussed above, but the other two have no obvious sequence relationship to the others, indicating different origins, but in each case they have the characteristic predicted secondary structure of 12 or 14 transdmembrane segments (TMS) [57] The 12 TMS topology for Wzx_B_ has been confirmed by gene-fusion analysis [58], It is interesting that WzxE is one of those with14 trans-membrane segments [59]. The differences in Wzx correlate with the general features of the O unit, which makes sense as Wzx simply translocates the O unit, and recently we showed that Wzx_B_ and Wzx_D_ are more efficient at translocating repeat units with a DDH side branch and that Wzx_E_ is more efficient at translocating repeat units without a side branch [59]. It seems highly unlikely that these highly divergent *wzy* and *wzx* genes diverged *in situ*, although apart from *wzy*_B_ at the old *rfc* locus, all are at the one *wzy* locus or the one *wzx* locus in the gene cluster.

### GC Content

When the LT2 gene cluster was sequenced [13] it was found that it could be divided into segments differing in GC content, all lower than that of typical *S. enterica* genes [13]. The *S. enterica* genomes completed to date typically average 51-52% GC. In contrast, the *abe*, *prt*, and *tyv* genes, and the GT, *wzx* and *wzy* genes have a very low GC content (28-37%). The *ddhDABC* genes generally have an intermediate GC content (~40-45%) as do the *manBC* genes (36-41%). The *rmlBDAC* genes also have an intermediate GC content (~40-51%), but it can reach the genomic average, although the group E *rmlC* gene is an exception at 33%. It is widely accepted that DNA segments of atypical GC content indicate horizontal transfer of genes from an ancestor of lower GC content [60,61]. The presence of segments of different GC content indicates different histories, either different sources or assembly over a period of time. All of the gene clusters under discussion have low GC content, which is interesting, as for published genomes high GC content is more prevalent [61].

We discussed above the variation in levels of divergence for *rml* genes and attributed it to movement of the gene clusters within between subspecies, such that *rmlB*, adjacent to the flanking housekeeping genes, generally varied with subspecies, as do the housekeeping genes, whereas *rmlC*, adjacent to O-antigen serovar-related genes, generally varies with O-antigen specificity. The same applies to the GC content of the *rml* gene set, with blocks of DNA closer to the O-antigen-specific genes having a lower GC content than those at the start of the gene cluster that vary with the subspecies and have the higher GC content. This suggests firstly, that there is nothing about these *rml* genes that makes it beneficial for them to have a low GC content in *S. enterica*, and conversely, that this supports the hypothesis that the genes transferred from another species.

It is interesting that in many but not all other polysaccharide gene clusters, the processing genes *wzx* and *wzy* have the lowest GC content, as is the case in the *S. enterica* gene clusters studied here, and this needs explanation. There have been reports that GC content drives codon usage of particular proteins however, the exact role and ramifications of these trends remains to be resolved [62,63].

### A Speculative Overview on Evolution of the Gene Clusters

Clearly the gene clusters we are discussing have evolutionary relationships, but because of the very different levels of gene divergence and the probable role of recombination, it is not possible to reach definitive conclusions on specific relationships. We have discussed above the striking fact that for genes that occur in two or more clusters, the gene order is maintained, and that this also applies to genes that carry out parallel but different functions. The explanation may be that these genes were in the same location and order in a common ancestral gene cluster and diverged *in situ*, or it may be that there is benefit in that gene order and new functions evolved by capture of those genes that then reached the appropriate locations in the gene cluster by selection. In this context it is interesting that in these gene clusters, the GT genes are in inverse map order to their function, and if selection is such that this will eventually be achieved after capture of a GT gene that replaces an existing gene, then that alone could explain *wbaO* and *wbaU* being at the same location in the gene clusters. Currently, we cannot distinguish between these hypotheses. In either case the changes involved are such that the timeframe for diversification of the 8 gene clusters must be such that much of it would have occurred prior to the divergence of *E. coli* and *S. enterica*. Perhaps the suite of gene clusters was obtained by *S. enterica* from outside by first gaining one, with the others then being obtained more readily by homologous recombination involving the shared genes at the two ends of the cluster.

**Figure 13:**
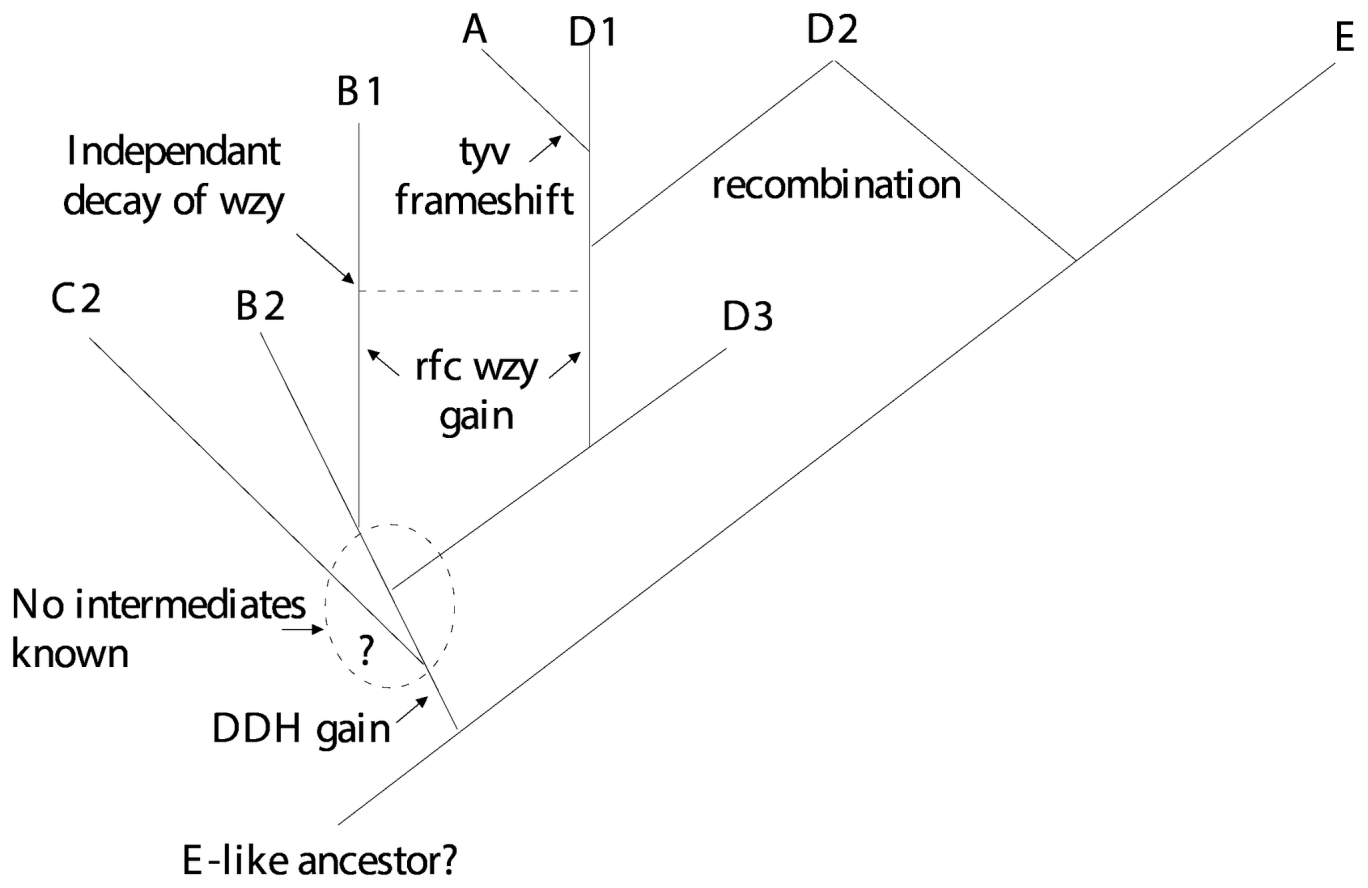
Model for evolution of the *S. enterica* Gal-initiated O-antigen gene clusters. The model is based on the assumption that the *ddh* genes were never present in group E. See text for details and other possibilities.

We present in Figure 13 what we consider to be a plausible scenario of how these gene clusters evolved, but it should be treated as a hypothesis that can be used as the basis for discussing group relationships.

#### Divergence of group E

We have placed the divergence of group E at the base of the tree using the assumption that it never had a DDH sugar sidebranch, and thus all of the other groups are united by having gained the DDH biosynthesis genes. The *ddh* genes and associated genes that distinguish the others from group E are in a block in the centre of the gene cluster. We discussed above the high-level sequence divergence in the *rmlAC* and *wbaN* genes flanking this central region. The differences are greatest at the boundary with the central region, and reach about 50%. If this reflects the time since the gain of the DDH sidebranch then it is indeed ancient. The gradient was proposed to be due to recombination during divergence between the lineages with and without the *ddh* genes [52], and this still seems the most probable explanation.

#### Divergence of group C2-C3

We have placed the next branch at the divergence of C2-C3 because it has a different main chain than the other O antigens, although having the same component sugars. This is a major difference and one could reverse the order of divergence for C2-C3 and E. We also suggest that the CDP-abequose pathway is the ancestral form of the related CDP-abequose and CDP-paratose/tyvelose pathways (Figure 3), as the abequose pathway is shared by the group B and C2-C3 gene clusters, and because the C2-C3 and B *abe* genes have undergone extensive divergence. This also puts the next node in our tree at the divergence of C2-C3 from the other DDH sugar-containing groups. The serovar-specific genes in the C2-C3 central region include all of the GT genes, as all of the linkages are unique to that group. The genes that appear to have been affected by flanking the central region are *abe* at the 5’ end and *manC, manB* and the 5’ end of *wbaP* at the 3’ end of the gene cluster (Figure 11). The divergence in the affected regions is 30 and 45% respectively and is lower than for the group E comparison, which fits the suggestion that divergence occurred after that of group E, but it is still indicative of a great age for this event. The affected regions cover about 1kb and 3kb of sequence at the 5’ and 3’ ends respectively, each with a consistent level of divergence, plus a region of a few hundred bases over which the divergence drops to the near zero level of the flanking DNA. Again the gradient is proposed to be due to recombination during divergence between the lineages, which in this case are those with and without the C2-C3 specific genes.

#### Divergence of groups D3 and B2

We come next to the divergence of the abequose- and tyvelose-containing groups. We discussed above the implications of the remnant *wzy* genes in groups B2 and D3 that are replaced functionally by a *wzy* gene at the *rfc* locus, and will return to that later.

Here we use the *wzx* and *wbaV* sequences, and the approach used above for groups E and C2-C3. The B1 and D1 *wzx* and *wbaV* genes differ in 33% of bases. They follow the *abe* gene and *prt/tyv* pair of genes, that are related to synthesis of CDP-abequose and CDP-tyvelose respectively. The *wzx* and *wbaV* genes presumably diverged after the establishment of the two alternative DDH sugars and the divergence level is similar to that seen in the C2-C3 comparison. It would appear that the abequose and tyvelose forms also diverged before the origin of *S. enterica*, and were acquired by that species more recently*.*

#### Derivation of groups B1 from B2 and D1 from D3

The derivation of B1 from B2 and of D1 from D3 must have involved gain of the a1-2 *wzy* gene at the *rfc* locus, followed by loss of function in the original *wzy* genes, and their subsequent independent decay. The same *wzy* gene at the same locus is involved with both B1 and D1 O antigen synthesis, and would have arisen only once, but at present it is not possible to say which of B1 or D1 arose first. In this case we have no clock to estimate the time frame, but we suggest that it takes thousands or more years for the observed degradation of the *wzy* genes. We are able to put a minimum period for presence in *S. enterica* of the 13 remaining genes (*rmlB* to *ddhC* and *wbaU* to *wbaP*) as the D3 genes have diverged to a similar level as for housekeeping genes in subspecies 1 and 2. The simple explanation which we prefer is that the ~4% divergence of these genes in our group D3 strain relative to the B1, B2, and D1 strains, arose during subspecies divergence, and on this hypothesis the group B and D gene clusters entered *S. enterica* before subspecies divergence, setting a minimum timeframe since that event. There are subspecies 2 strains for all of these groups and further study may reveal the subspecies 2 sequence for groups B1, B2 and D1.

#### Origin of group D2

The origin of D2 by recombination between the group E and D1 gene clusters is reasonably clear. The 5’ end of the D2 gene cluster is near identical to that of group D1, and the 3’ end to that of group E, so the H-repeat element at the junction may well have been involved in mediating the recombination event, as proposed in Figure 7. However, all three serogroups are quite common and the two segments of D2 shared with E and D1 would undergo random genetic drift as a single entity, rather than separately for the gene clusters involved, so the level of divergence may not reflect the age of D2. The nature of the recombination event is also complicated by the presence of a similar H-repeat remnant in D2 and D3 (that is not found in D1), and an apparent *wzy* remnant that is common to the D1, D2 and D3 strains studied.

#### Multiple origins for group A

The most recent node on our tree relates to the origin of group A. Only the Paratyphi A serovar has been studied in detail, and a single base change served to prevent conversion of CDP-paratose to CDP-tyvelose, and this seems to be all that is necessary to generate a group A strain, as the other genes are unchanged, although there is also a replication of a short segment of the gene cluster. Each of the 4 group A serovars appear to have arisen independently, as there are 4 group D1 serovars in the KW scheme that have the same H antigens and so are putative ancestors.

### concluding remarks

In this paper we have looked at the relationships of the eight Gal-initiated O antigens in *S. enterica*, and in Figure 13 present a putative phylogenetic tree for the gene clusters. One of the strategies that we used to generate the tree is to take regions of two or more gene clusters that are shared but which are quite divergent. These shared but divergent segments are all between the central regions containing different genes in the groups being compared, and the more distal shared segments that are near identical. We have used the level of sequence divergence across such shared segments to estimate the time for the co-existence of the adjacent sets of group-specific genes. It seems reasonable to attribute the level of divergence in these shared segments to the time since their most recent common ancestor, but how reasonable is the assumption that the divergence occurred while these diverging sequences were associated with the adjacent sets of group-specific genes? Although we have made this assumption, it is a difficult question to answer. The alternative is that the two segments were brought together more recently, but we consider that less likely given the maintenance of gene order across the gene clusters.

Seven of our O antigens have a DDH side branch residue, but although such sugars are very rare this situation is not unique as there is a parallel set in *Y. pseudotuberculosis* (see 44,64,65. Most of the *Y. pseudotuberculosis* O antigens include a DDH side branch. In *Y. pseudotuberculosis* the CDP-DDH pathway can lead to abequose, paratose or tyvelose in the O antigen, as in *S. enterica*, but also to ascarylose and two DDH-related sugars: yersiniose (A), which is a DDH with a 2-carbon addition, and L-altrose, a 6-deoxy sugar that utilizes only the first half of the CDP-DDH common pathway. Of interest here is that the gene patterns for the CDP-pathways in the two species are identical with the location and order of the *ddhDABC*, *abe* or *prt*/*tyv*, the DDH transferase, and the *wzx* genes being the same in both species. The sugar-specific genes for ascarylose, yersiniose (A) and L-altrose also fit the same general pattern. However, there is substantial sequence divergence between the gene clusters of the two species, suggesting that the gene order observed is ancient and would have been present even before most of the events discussed in this paper. It seems then that this organization is very ancient and is even better developed in *Y. pseudotuberculosis*. However, it has not been reported in other *Yersinia* species and again we have a diversification of this pathway in only one lineage of the genus. *Y. pseudotuberculosis* also has several alternative forms of the O-antigen main chain and all are WecA-initiated and so in this respect differ from the situation in *S. enterica.*

We should also point out that while the tree shown in Figure 13 is speculative in some of its detail, there is strong support for the galactose-initiated O antigens having a history such as that shown, with the ambiguity lying mostly in the early branching pattern.

We have focused much of our work on *Salmonella* because the existence of a group of similar structures that can be shown result from divergence in shared genes enabled us to make deductions about their evolution and the timeframe. There is no reason to believe that these O antigens are unusual in any other way, and similar evolutionary processes and timeframes probably apply to many or all of the variable surface polysaccharides.
